# Nanostructure domains, voids, and low-frequency spectra in binary mixtures of *N*,*N*-dimethylacetamide and ionic liquids with varying cationic size†

**DOI:** 10.1039/c9ra09041j

**Published:** 2020-01-08

**Authors:** Th. Dhileep N. Reddy, Bhabani S. Mallik

**Affiliations:** Department of Chemistry, Indian Institute of Technology Hyderabad Kandi-502285 Sangareddy Telangana India bhabani@iith.ac.in +91 40 2301 6032 +91 40 2301 7051

## Abstract

Classical molecular dynamics (MD) simulations were carried out on binary mixtures of *N*,*N*-dimethylacetamide (DMA) with hydroxide based ammonium ionic liquids (ILs), tetraethylammonium hydroxide (TEAH), tetrapropylammonium hydroxide (TPAH), tetrabutylammonium hydroxide (TBAH), at three different mole fractions of IL (*X*_IL_). The solvation of DMA molecules by ions of ILs was studied by the combined distribution function (CDF). CDFs show that anions have strong correlations with the DMA due to the hydrogen bonding. Increasing the DMA disrupts the nanosegregated domains and causes changes in correlations of cation–DMA and anion–DMA. Also, increased translational motion of ions, as well as the fluidity of IL and a significant improvement in self-diffusion coefficients, are observed with the presence of more DMA. The structural microheterogeneity was investigated using the Voronoi tessellation method. Domain analysis confirms the formation of discreet domains by anions at all the mole fractions. The results also complement the experimental observations, which suggest that two types of aggregations are possible in given mixtures: below and above 0.5 *X*_IL_. When the alkyl chain length on the cation increases, a notable decrease in ion translational motion was observed in the IL rich region. In the concentrated IL mixture, the self-diffusion coefficient of the cation is higher than that of the corresponding anion; further addition of IL (*X*_IL_ < 0.5) results in weaker interactions between DMA and anion when compared to DMA–cation. The mean collision time of each species is found to have an inverse relation with *X*_IL_. The analysis of the vibrational density of states provides the low-frequency spectral feature of the mixtures.

## Abstract 2

Classical MD simulations were carried out on binary mixtures of DMA with hydroxide based ammonium ILs, TEAH, TPAH and TBAH, at three different mole fractions of IL.

## Graphical abstract

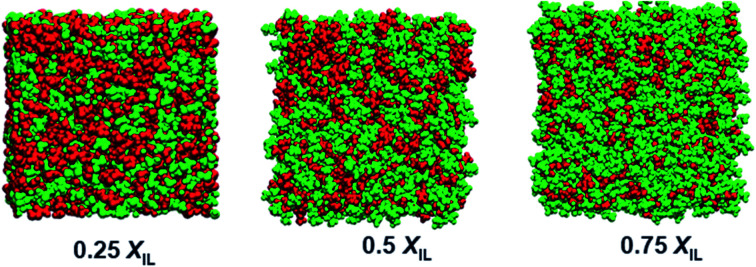

## 1. Introduction

ILs are considered to be novel solvents, because of their wide variety of desirable properties and promising applications.^1–5^ Tuning the ions of ILs can lead to a significant change in physicochemical properties. ILs show excellent thermal and electrical stability.^6–9^ The knowledge of interactions between ILs and molecular solvents is essential for industry and research^10–12^ for designing a group of alternative solvents for various applications. Addition of molecular solvents to ILs strongly affects various properties such as density, viscosity, and conductivity. Many room temperature ILs (RTILs) are non-volatile, non-flammable, non-explosives, and can be recycled. Ammonium based ILs are considered to be very important in a wide range of applications in chemical and biochemical processes.^1,5,10,11^ Alkylammonium-based ILs have been studied well.^6,13–16^ Trialkylammonium ILs were extensively studied by Pott and Méléard^17^ using X-ray scattering experiments, and by Shimizu *et al.*^18^ using molecular dynamics (MD) simulations. These studies reveal that the change in alkyl chain length strongly affects the structure, but it does not affect the intermediate and high-*k* peaks^17,18^ obtained from X-ray experiments. Both experimental and computational studies have confirmed the presence of nanoscale segregation. Quaternary ammonium (QA) ILs exhibit exciting properties. For example, QA ions combined with (bis(trifluoromethylsulfonyl)imide) [NTf_2_]^−^ anion showed a wide electrochemical window, better chemical, and thermal stability when compared to corresponding pyridinium and imidazolium ILs.^19,20^ Applications of these ions were well known in nanowire-based lithium-ion batteries.^21–24^ Among the ammonium-based cations, tetraalkylammonium (TAA) ions^25^ cations are considered to be hydrophobic, and the series of small symmetric TAA cations were studied to investigate the effect of hydrophobicity on ion hydration.^26–28^ The hydrophobic character can be tuned by changing symmetry and length of the alkyl chains. Tetraethylammonium (TEA), tetrapropylammonium (TPA) and tetrabutylammonium (TBA) cations were studied extensively.^29^ However, the correlation between thermodynamic and structural data is not well understood. Combining the TAA based ILs with the molecular solvents would yield attractive and desired properties for various applications. Among the many organic solvents, acetamide based molecular solvents are essential due to the presence of –C=O and –NH– groups, which are also present in other biomolecular molecules. Studying the mixture of ILs and acetamide molecules will be useful to understand intermolecular ionic interactions with biomolecules, and furthermore, they are important solvents in industry and research. DMA has high polarity, which made it soluble in a wide variety of polar and nonpolar solvents and can be used to get insights of dipole–dipole and ion–dipole interactions.^30–34^ The solvation of DMA with ammonium based ILs can be considered as one step forward in understanding the solvation of complex bio molecules in ILs.

In this work, the structure and dynamics of the binary mixtures of three tetraalkylammonium hydroxide ILs and DMA were investigated by molecular dynamics simulations. The TEA, TPA, and TBA cations are chosen so that changes in structure and dynamic properties can be calculated with the systematic increase of the cationic chain length. It is well established that the change in alkyl chain length has a significant impact on segregation, diffusion, and domain formation.^15,16,35–37^ The simulations were further analyzed to get insight into the domain formation. The DMA molecules show different domain counts in different mixtures. To the best of our knowledge, articles published on mixtures of ammonium-based ILs with DMA are minimal.^38–41^ The present work is aimed at understanding the interactions between ILs and molecular solvent, DMA, at an atomic level description.

## 2. Computational methodology

**Fig. 1:**
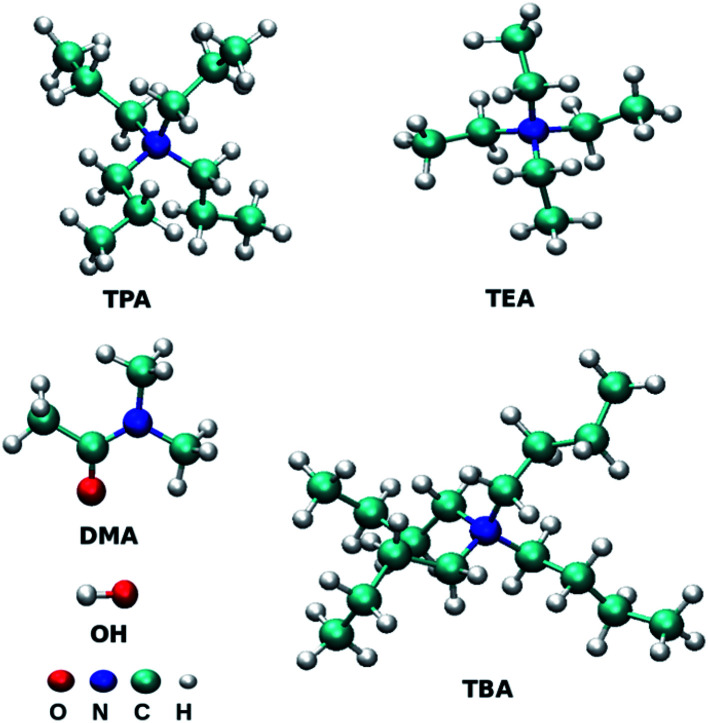
Structures of molecules or ions TEA (tetraethylammonium), TPA (tetrapropylammonium), TBA (tetrabutylammonium), OH (hydroxide) and DMA (dimethylacetamide). The structures were prepared using the VMD programs.^97^

All-atom molecular dynamics simulations were performed for binary mixtures of three tetraalkylammonium ILs with DMA solvent at three mole fractions of ILs using GROMACS 5.0.4.^42,43^ The total number of molecules was fixed to 500, and the ion pairs were replaced by the DMA molecule according to the target mole fractions of ILs. Initial geometries of ions were optimized by the Gaussian software^44^ package at the B3LYP/6-311+G(2d,p) level. Restrained Electrostatic Potential (RESP) method^45^ was used to derive the partial charges of atoms at B3LYP^46,47^ electronic structure level using 6-311+G(2d,p) basis set. The partial charges of the atoms are assigned using the antechamber package.^48^Fig. 1 presents the cations and anion used in this study. The *xyz* format files of optimized structures are given in ESI.† Packmol software was used to generate the initial configuration of 500 chemical entities.^49^ Non-bonded parameters for cations of IL were taken from the work of Chang *et al.*,^50,51^ and the force field parameters for DMA are directly taken from Optimized Potential for Liquid Simulations all-atom (OPLS/AA) force field.^50^ Non bonded force field parameters are given in Table S1 of ESI.† The generated initial structures of each mole fraction were further subjected to energy minimization using the steepest descent algorithm.^52^ LINCS algorithm was used to constrain all the bonds associated with the hydrogen atom.^53^ Surface effects were eliminated by applying the periodic boundary conditions in all three directions. The system was heated at 798.15 K for two ns to mix the ions randomly. The final configuration was used further in the stepwise cooling procedure. In cooling procedure, the temperature was systematically decreased by 50 K for every 200 ps and cooled down to room temperature in 2 ns. Simulation time of 20 ns in *NpT* ensemble was performed to reach the equilibrated density. Density was calculated from the further 30 ns simulation in the *NpT* ensemble (Table S2†). For all the mixtures, the density increases with an increase in the concentration of IL. The temperature was controlled using the V-rescale thermostat,^54^ and the coupling constant was 0.1 ps. Berendsen barostat^55^ was used to control the pressure in isothermal-isobaric simulations with a coupling constant of 2.0 ps. Equilibration for ten ns was performed in the NVT ensemble. Nonbonded interactions were calculated using the cut-off value of 1.2 nm. 1 fs time step was used during the production run. Long-range electrostatic interactions were treated with the Particle Mesh Ewald (PME) switch method with the cut-off value of 1.2 nm.^56^ The simulations in the NVE ensemble were performed for 200 ns to calculate the properties from the obtained trajectories.

## 3. Results and discussion

**Fig. 2:**
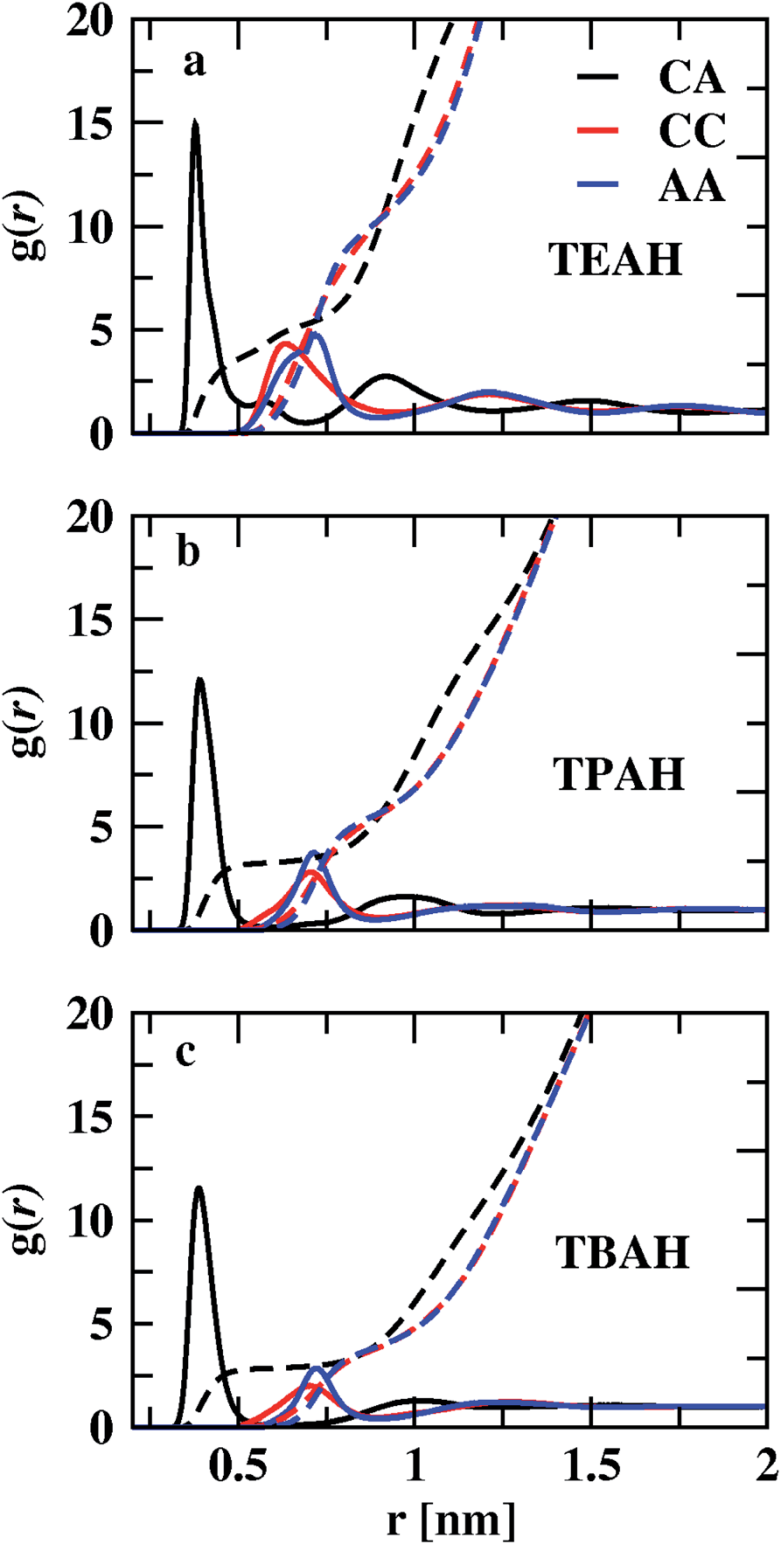
Centre of mass RDFs for cation–cation (CC), anion–anion (AA) and cation–anion (CA) for three different ILs TEAH (a), TPAH (b), and TBAH (c) at 0.25 *X*_IL_. The CA interactions are stronger when compared to CC and AA. The interactions between cation and anion tend to low when cation has larger head group.

We studied the intermolecular interactions between ammonium-based ILs (TEAH, TPAH, and TBAH) and DMA molecules using the radial distribution functions (RDFs). The RDFs between the center of masses (COMs) of cation and anion are shown in Fig. 2, S1 and S2.†Fig. 2 shows the influence of the size of the cationic group on the structure at 0.25 mole fraction of IL. The order of the positions of first peak of cation–anion (CA) RDFs is TEAH (0.380 nm) < TPAH (0.390 nm) < TBAH (0.391 nm). Due to strong electrostatic interactions between small ions as well as less steric hindrance, ions are closely packed in TEAH. In TPAH and TBAH, the cations are relatively larger due to which the positions of the first peaks of cation–anion (CA) RDFs appear at larger distances than in TEAH. The height of the first peak also decreases with increasing alkyl chain length of the cation. The heights of the CA RDFs for 0.25 mole fraction of IL are as follows: TEAH (14.50) < TPAH (11.90) < TBAH (8.44). Hydrophobicity of the TPAH and TBAH is higher than the TEAH due to larger alkyl chains.^57^ Therefore, the electrostatic interactions between cation and anion tend to low when the cations possess the larger alkyl chains. Experimental and quantum chemical calculations also show similar results.^57^ TEAH IL has a regular 3D packing of ions which diminishes the DMA–IL interactions.^57^ The interionic interactions within the IL depend on the alkyl chain length of the cation. Cation–cation (CC) and anion–anion (AA) interactions are weak when compared to CA. TEAH shows a higher degree of charge ordering than the other two ILs, which is due to higher electrostatic interactions between opposite ions. We show the charge density distribution in Fig. S3.† In all the mixtures TEAH showed the higher charge density than other two ILs. Also, it can be observed that the oscillation of CC and AA RDFs are out of phase with CA RDFs. The ionic nature of ILs tends to form local charge ordering. TEAH shows the local charge ordering over multiple solvation shells as compared to TPAH and TBAH. The bigger size of cations in TPAH and TBAH hinders the approach of anions toward them; this causes a decrease in electrostatic interaction between opposite ions. The out of phase behavior is a result of the occupation of counterions around central ion in the first solvation shell, which can be called as ion cage. Similar observations were found in earlier studies.^58–66^ This order is also explained based on the pseudo lattice arrangement of liquids.^67,68^ The presence of the same charged ions in the first solvation shell is minimal. From Fig. 2, a significant assembly of hydroxide anions occurs around cations with an average CA distance of 0.39 nm. The decrease in the height of the first peak with the increase in the alkyl chain length of the cations is one of the main features in these mixtures.

The coordination numbers, calculated by integrating the RDFs to the first minimum, are given in Table S3.† The CA coordination number in TEAH is 2.87. Three anions are present in the first coordination shell of the cation in TEAH of 0.25 mole fraction of IL. Moreover, for other ILs at the same mole fraction, the coordination number decreased to 2.42 (TPAH) and 2.14 (TBAH). This again indicates the decrease in electrostatic interactions between counter ions of IL with increasing alkyl chain length and steric hindrance. Three anions can approach the cation at the same time in TEAH, which is not possible in TBAH and TPAH ILs due to steric hindrance and hydrophobicity. The impact of cation chain length is very less after the first solvation shell in all three mixtures and at all the mole fractions of IL.

CC and AA interactions are weaker than CA interactions. Strong electrostatic interaction exists between cation and anion. Cations and anions form long-lived clusters. Ion pair lifetimes were calculated using TRAVIS software and are included in Table S4.† All the ILs show lifetimes in the scale of nanoseconds, which show the presence of long-lived clusters. TEAH shows the lowest ion pair lifetime at 0.25 mole fraction of IL. The height of the first peak of cation–cation RDF decreases from TEAH to TBAH. The first peaks of cation–cation RDFS also move towards higher distances with increasing alkyl chain length. The second peak of cation–cation RDFs appears around 1.2 nm. The long-range charge ordering is present between cations. The anion–anion (AA) RDFs show the second peak at 1.2 nm. For the anion–anion RDF, the first peak shows a similar order as CC RDFs. Even though the size of the anions is smaller than the cations, the first peaks of the AA RDFs are at more considerable distances. In both CC and AA RDFs, the considerable influence of alkyl chain length is observed on the first peaks. This reflects the reduction of the ability to coordinate more ions when alkyl chain length increases. The comparison of RDFs for 0.5 and 0.75 *X*_IL_ are shown in Fig. S1 and S2,† respectively.

**Fig. 3:**
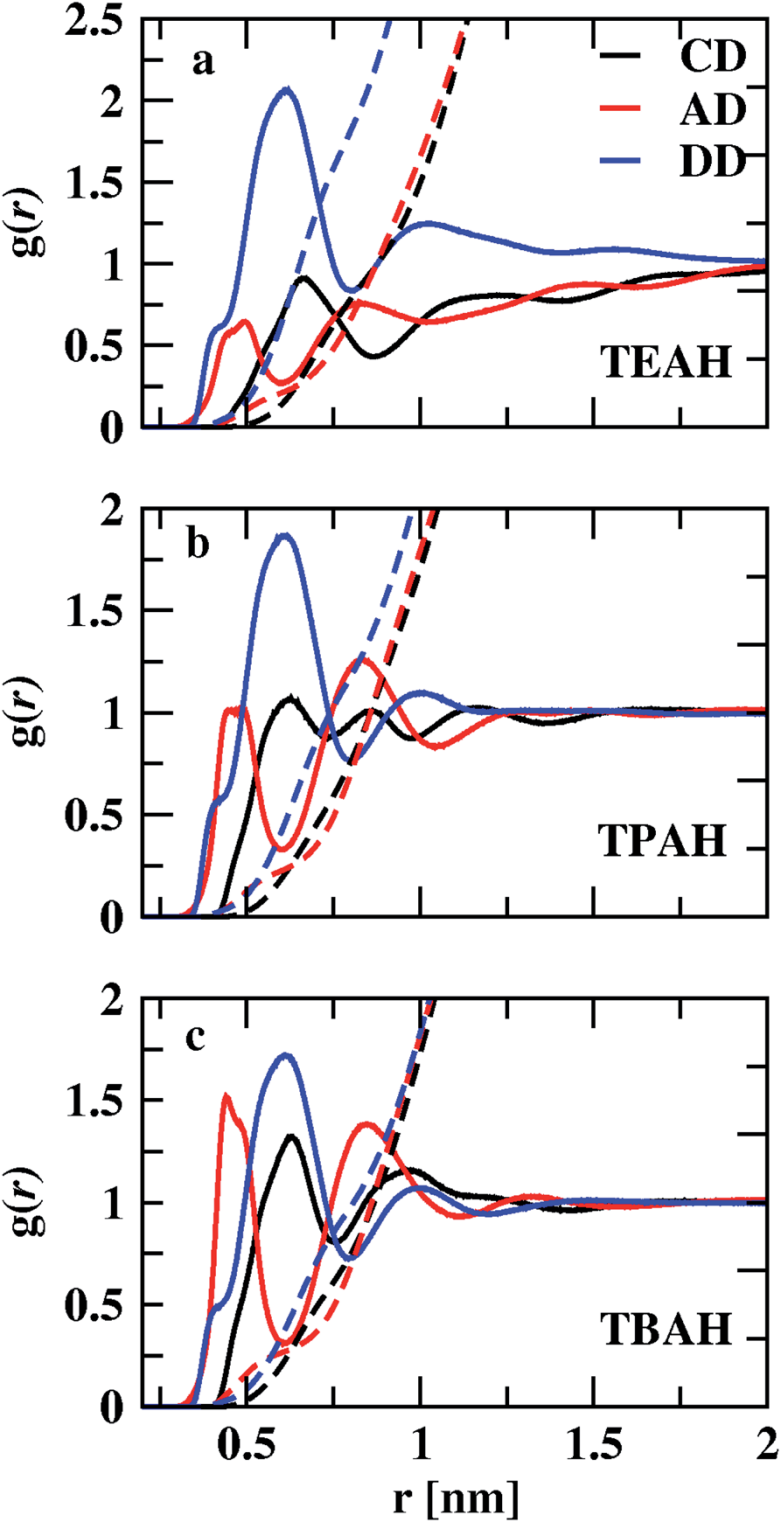
Comparison of the centre of mass RDFs between cation–DMA, anion–DMA and DMA–DMA for three different ILs TEAH (a), TPAH (b) and TBAH (c) for 0.25 mole fraction of IL. The AD interactions are found to be at lower distances than CD and DD interactions. Anions form the hydrogen bonding with the DMA molecule and show strong interaction.

**Fig. 4:**
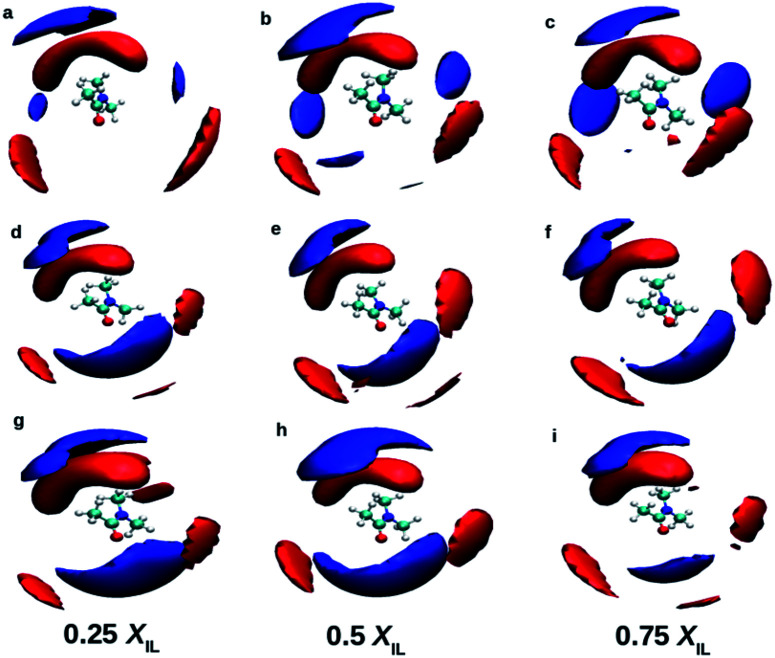
Spatial distribution functions of the cation (blue) and anion (red) around the DMA molecule in mixtures at different mole fractions. Top, middle and bottom rows indicate TEAH, TPAH and TBAH mixtures with DMA, respectively. The distribution of cations around DMA is different in TEAH + DMA mixture. Anion shows similar distribution around DMA in all the mixtures.

Fig. 3 shows the RDFs between cation–DMA (CD), anion–DMA (AD) and DMA–DMA (DD) for 0.25 mole fraction of IL. Figures for 0.5 and 0.75 *X*_IL_ can be found in ESI (Fig. S4 and S5†). The most notable effect in Fig. 3 is out of phase behavior of AD RDFs with DD RDFs. Overall, DMA molecules interact more with themselves rather than with cation or anion. Similar results were observed from the experiment.^57^ In the mixture of TEAH and DMA, DD RDFs show out of phase oscillations with AD RDFs. The same feature is observed in the other two ILs. The first peak of DD RDF has more height than CD and AD RDFs in all the mixtures. The DD and CD RDFs show first minima at a larger distance than AD RDFs. For example, the first peak of DMA–DMA RDF in TEAH is found at 0.62 nm, and cation–DMA is at 0.68 nm, whereas AD is found at 0.51 nm. The anion comes close to the DMA molecule when compared to cation and DMA due to the small size of the anion. The ion-induced dipole interactions also play an important role in this interaction. The anions form hydrogen bonding with the carbonyl oxygen of the DMA molecule.^57^ This interaction is present at all mole fractions of IL. Shoulders are observed for DD RDFs at 0.42 nm in all the ILs. This is due to the presence of anions in the voids of DMA and forms H-bond interactions. This can be observed from the AD RDFs. The AD RDFs show the first peak at 0.42 nm, which is the position of the shoulder in DD RDFs. Another interesting feature is that DMA interaction with cation, anion, and DMA itself. DMA interaction with cation is high in TBAH and low in TEAH. The same is observed for AD RDFs. A significant increase in the correlations between anion and DMA is observed from TEAH to TBAH. The hydrophobic interactions between DMA and IL alkyl groups are relatively stronger when cation has a large head group. The bulky nature of cation does not allow the anion to come close in TBAH. As a result, the increase in anion–DMA correlations can be observed. However, when we see DD RDFs, the interaction between DMA molecules is high in TEAH, which is opposite to the former case. The diminished intensity of the first peak of DD and AD RDFs from TBAH to TEAH reflects the increased correlations between DMA molecules. This leads to the formation of DMA clusters in TEAH. The COM RDFs include all the interactions contributed from alkyl chains and charged nitrogen atom with cation. It is difficult to characterize the specific interactions and the direction of approach of DMA and anion of IL from COM RDFs. For this purpose, we have calculated the combined distribution functions (CDFs) in later sections to further investigate the obtained results from COM RDFs. Comparison of similar interactions in different mole fractions is presented in Fig. S6–S8. † Monotonic decrease of the first peak of RDFs between cation and anion (CA) is observed upon addition of IL to the mixture. Enhanced correlations between the ions at a lower concentration of IL causes this decrease in first peak height with IL concentration. The height of the second peak also decreases with the addition of IL. CC and AA RDFs also show a similar trend as CA RDFs. This reflects a decrease in correlations between ions. Peak positions are invariant with the addition of IL. The first peak positions of CA, CC, and AA RDFs, are found at 0.38, 0.65, and 0.73 nm, respectively. These peak positions illustrate that CA correlations are the strongest interactions. Cations interact with themselves through van der Waals forces between alkyl side chains. Bhowmik *et al.*^25^ observed the decrease in interaction between cation and anion in aqueous solutions of tetraalkylammonium (TAA) ILs with an increase in IL concentration. A monotonic increase in the height of the first peak is observed with the addition of IL for CD, AD, and DD RDFs. The clustering process of ILs around the DMA is more feasible at a higher concentration of IL. A progressive weakening of interaction is observed between ions and DMA in the first solvation shell with a decrease in IL concentration. As discussed earlier, the first peaks of anion–DMA (AD) RDFs are at lower distances than cation–DMA (CD) and DMA–DMA (DD) RDFs. The effect of concentration is more in TEAH + DMA when compared to TPAH + DMA and TBAH + DMA mixtures. The heights of the first peaks change more in TEAH + DMA mixture for CD, DD, AD, CA, CC, and AA RDFs. The screening effect is present in the TBAH mixture because of the long alkyl chain around the charged center. The change in interactions is less with the addition of IL. This difference between interactions in mixtures is associated with different types of interactions between IL and DMA. The visual inspection of ions around DMA would give more clarity about the structural arrangement of ions around DMA molecules. We calculated the spatial distribution functions (SDFs) of ions around the DMA using TRAVIS^69^ software. SDFs of cation and anion around the DMA are shown in Fig. 4. In all the ILs, anion clouds are found to be in the opposite to the oxygen atom of DMA. The negative charge on anion has a repulsive interaction with the carbonyl group of DMA. This leads anion to occupy the rear side of the carbonyl oxygen atom. In TEAH, the distribution of cations is found to be around the nitrogen atom of the DMA molecule as contrast to the case for TPAH and TBAH mixtures; the distribution cloud of the cation is situated around the carbonyl oxygen atom of DMA. Strong electrostatic interaction is present between cation and anion in the TEAH mixture. The cation of TEAH IL interacts less with DMA molecules than TPAH and TBAH ILs. The alkyl chains of cation and methyl groups of DMA interact more when the cationic head group is larger. Cation clouds are found around anion.

**Fig. 5:**
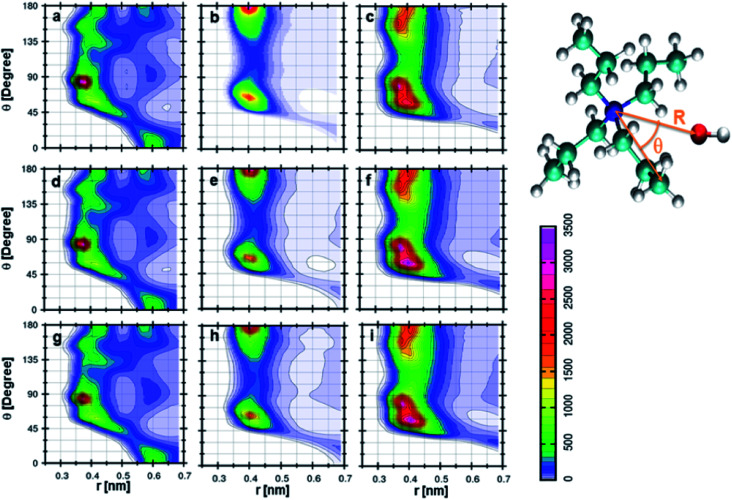
Combined distribution functions between cation and anion at different mole fractions. (a–c) Represent the 0.25 *X*_IL_, (d–f) represent the 0.5 *X*_IL_ and (g–i), represent the 0.75 *X*_IL_. Left, middle and right columns depict the TEAH, TPAH and TBAH mixtures with DMA respectively. Definition of angle *θ* and distance *R* between cation and anion are shown in a model represented in CPK style.

The orientation of cation, anion, and DMA geometries around each other can be further investigated by calculating the combined distribution functions (CDFs). The relation between angle and distances can be established through combined distribution functions (CDFs), which were calculated by combining the radial distribution functions (RDFs) and angle distribution functions (ADFs).^69^ The CDFs between cation and anion are shown in Fig. 5. Fig. 5a represents the CDF of the cation–anion pair in 0.25 mole fraction of TEAH. This CDF shows the most probable configurations at 0.37 nm, which correlates to the most probable distance of anion from the nitrogen atom of TEA cation. The corresponding angle between cation and anion is close to 90°. Fig. 5d and g, which represent different mole fractions, also show similar behavior. The coordination numbers reveal that three anions symmetrically surround the first solvation shell of the cation. These three anions occupy the voids which are present between the alkyl chains of the cation. Fig. 5b indicates the CDF at 0.25 mole fraction of TPAH. TEAH shows only one most probable distribution for anion around cation, but in TPAH two distributions are found. TPAH shows two distinguishable contours at two different angles (65° and 180°) and the same distance, 0.4 nm. The maximum of first peak values is found at 0.4 nm. The peak maximum of RDF moves from 0.37 nm in TEAH to 0.40 nm in TPAH. Anions are at a slightly larger distance from the nitrogen atom of the cation in TPAH. These peaks reflect the symmetric structure of anions around cations. This means all the oxygen atoms of anion which approach the cation are found at the same distance. The flexibility of side chains with increasing the alkyl chain length allows the anions to approach cation in different directions. It is also interesting to mention that TPAH shows a coordination number close to TEAH, which is around three. TPAH has a slightly lower coordination number than TEAH due to the steric hindrance in TPAH. The cation hinders the anion from coming close when it has a larger alkyl chain. Fig. 5e and h also related to TPAH at 0.50 and 0.75 mole fraction of IL, respectively, and show similar CDFs. Fig. 5c shows the CDF between cation and anion at 0.25 mole fraction of TBAH, which has a different pattern than TEAH and TPAH. This CDF has three different contour distributions at three values of angles. The distance is found to be slightly different for different distributions. The preferred distance for these distributions is around 0.4 nm. This represents a configuration where the oxygen atoms of the anion approach the nitrogen atom of the cation in three different angular directions. These contours are observed at 60°, 80°, and 180°. As the alkyl chain length increases from TEAH to TBAH, the flexibility of side chains increases; this flexibility facilitates the anions to approach in different directions. The symmetry of alkyl chains decreases around the central atom when the cationic head group has larger size. Among all the three peaks, the peak around 60° is prominent. The most probable angle for anion to approach the cation is 60°. The other factor is a steric hindrance, which increases with the side chain. This factor limits the number of anions around the cation. This can be observed from the coordination numbers. The cation–anion interactions can be tuned by changing the cationic head group in IL + DMA mixtures.

**Fig. 6:**
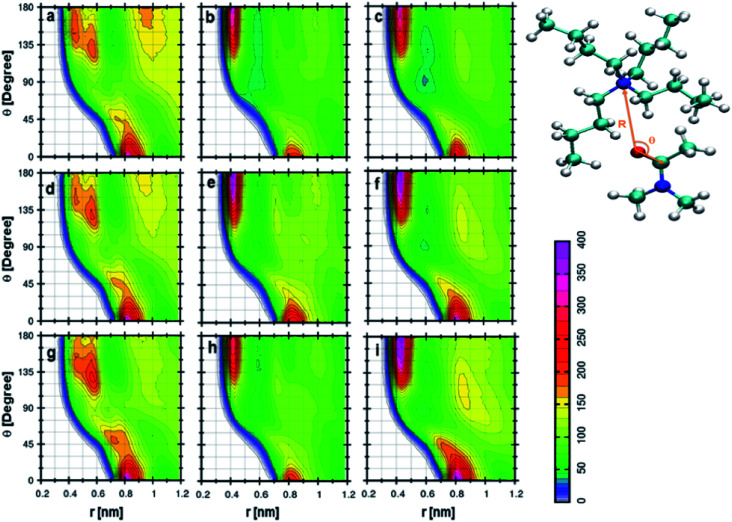
Combined distribution functions between cation and DMA at different mole fractions. (a–c) Represent the 0.25 *X*_IL_, (d–f) represent the 0.5 *X*_IL_ and (g–i), represent the 0.75 *X*_IL_. Left, middle and right columns depict the TEAH, TPAH and TBAH mixtures with DMA respectively. Definition of angle *θ* and distance *R* between cation and anion are shown in a model represented in CPK style.

Fig. 6a indicates the CDF between cation and DMA in 0.25 mole fraction of TEAH. If the angle is higher than 100°, the preferred distance between the oxygen atom of DMA and nitrogen atom of the cation is between 0.5 to 0.6 nm. One more most probable distribution is observed at 0° angle and 0.85 nm distance. The most probable distance shows the existence of long-range interactions. This distribution depicts the interaction of the DMA molecule towards the cation. The peak at 0.85 nm shows strong interaction than the other at 0.6 nm. This is due to the steric hindrance of ethyl chains which have low flexibility and form more symmetric spherical type arrangement around the central atom. The distribution of DMA molecules around cation is not symmetrical like anion. Fig. 6d and g are for 0.5 and 0.75 mole fraction of TEAH in DMA. The peak maximum at 0.85 nm slightly increases with the mole fraction of IL due to an increase of IL concentration. Mole fraction of IL alters the DMA–cation correlations. Fig. 6b indicates the CDF between cation and DMA in 0.25 mole fraction of TPAH. In this CDF two distinguishable peaks in RDFs are observed. One peak is around 180° and 0.45 nm, and the other one is around 0° and 0.8 nm. The peak around 180° shows a higher intensity than the peak at 0°. van der Waals interaction increases between alkyl chains of cation and methyl groups of DMA from TEAH to TBAH. Here, the DMA molecule approaches the cation in the opposite direction of the alkyl chain, which is similar to TEAH. A similar type of behavior can be observed in Fig. 6e and h, which indicate the 0.5 and 0.75 mole fractions of TPAH in DMA, respectively. Fig. 6c shows the CDF between cation of TBAH and DMA. This is similar to the case of TPAH. This is obvious that when alkyl chain length increases, the DMA molecule tends to approach the cation in between the alkyl chains. The same is true for Fig. 6f and i corresponding to 0.5 and 0.75 mole fractions of TBAH in DMA, respectively. Overall, these CDFs reveal that DMA molecule preferentially occupies two positions around cation in all the ILs. TEAH shows low probability around 180° as compared to TPAH and TBAH. This increase is attributed to the steric hindrance of alkyl chain with a higher number of carbon atoms. Experimental results also revealed that TEAH behaves differently towards the DMA as compared to TPAH and TBAH.^57^ The CDFs from above discussions also show that the solvation of DMA is different in TEAH + DMA mixture as compared to TPAH + DMA and TBAH + DMA mixtures.

**Fig. 7:**
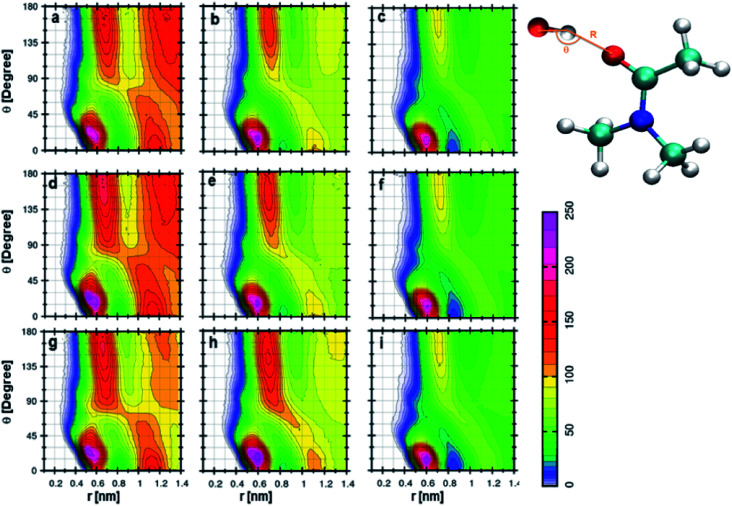
Combined distribution functions between anion and DMA at different mole fractions. (a–c) Represent the 0.25 *X*_IL_, (d–f) represent the 0.5 *X*_IL_ and (g–i) represent the 0.75 *X*_IL_. Left, middle and right columns depict the TEAH, TPAH and TBAH mixtures with DMA, respectively. Definition of angle *θ* and distance *R* between cation and anion are shown in a model represented in CPK style.

Anions show a different type of CDFs when compared to cations. Fig. 7a indicates the CDF between anion of TEAH and DMA molecule. This CDF shows several probable distributions because of long-range interactions. This feature is already observed from the RDF of anion–DMA. The anion can be found around carbonyl oxygen of DMA due to hydrogen bonding.^57^ The interaction between the anion of IL and DMA becomes stronger with the increase of IL concentration. The redshift was observed in the wavenumbers obtained from FTIR spectra when the concentration of IL increases form low to high.^57^ Similar results in CDFs are observed in Fig. 7d and g, which correspond to 0.5 and 0.75 mole fraction of TEAH, respectively. In Fig. 7b, CDF between anion of TPAH and DMA is shown for 0.25 *X*_IL_. The most probable contour distribution in CDF of TPAH resembles TEAH in Fig. 7a. In the case of TPAH, probable distributions are due to the lack of long-range interactions. The bulky alkyl chains screen the long-range interactions between anion and DMA. The anion also approaches DMA from the backside of C=O. Fig. 6e and h also show similar CDF as Fig. 6b. The SDFs of anion around the DMA molecule also show the distribution of anion around the carbonyl carbon of DMA. TBAH shows the most probable distribution around 0.6 nm, which is shown in Fig. 7c, f and i. The anion interacts with DMA molecules more in TEAH rather than TPAH or TBAH. As we discussed earlier, the increase in alkyl chain length restricts the anion to facilitate the long-range interaction. From TEAH to TBAH, the long-range interaction disappears due to the increased bulkiness of cation, which also affects the anion–DMA correlations. Quantum chemical calculations showed that the interaction between IL and DMA followed the order of TEAH > TPAH > TBAH.^57^ To get a better description of structural arrangements in a given mixture, we have applied the Voronoi tessellation analysis.^69,70,70^ Voronoi tessellation method gives quantitative information about domains formed by the defined subunit.

### 3.1 Micro-heterogeneity in IL + DMA mixtures

**Fig. 8:**
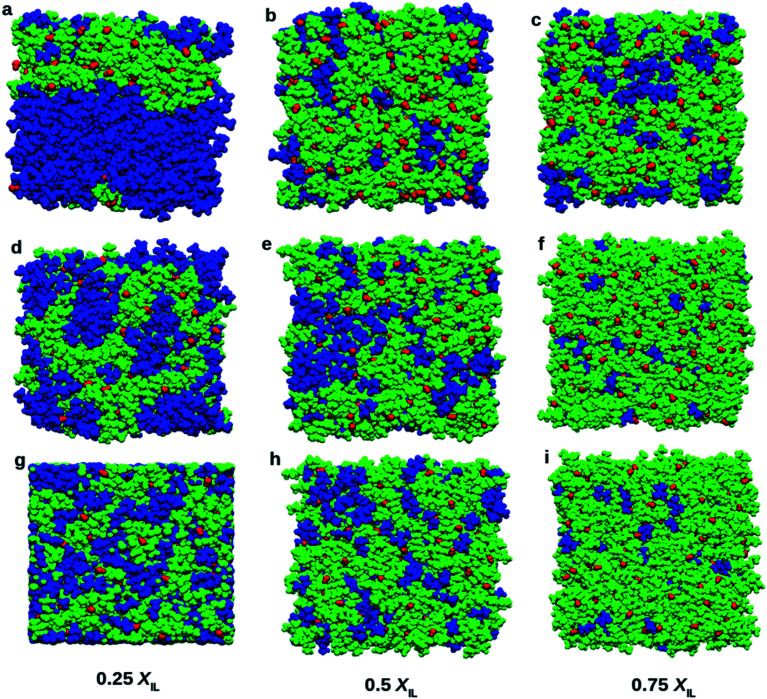
Snapshots of boxes at different mole fractions of ILs. Blue, red, and green colors indicate DMA, hydroxide anion, and cation, respectively. Top (a, b and c), middle (d, e, and f) and bottom (g, h, and i) rows show the mixtures of DMA with TEAH, TPAH, and TBAH, respectively. DMA molecules tend to form two types of clusters. At low *X*_IL_, large clusters of DMA molecules were found. As the concentration of IL increases the DMA molecules tend to break into small clusters.

Visualization of simulated boxes gives us some crucial information about micro-heterogeneity. Fig. 8 shows the snapshots of simulated systems at the end of the production run. We have performed two types of domain analysis. First, we considered cation, anion, and DMA molecules as different subunits of the systems. Segregation of DMA into a single domain can be observed in Fig. 8a, corresponding to 0.25 mole fraction of TEAH. Fig. 8b and c also show continuous domains. The self-aggregation of DMA molecules is more at higher mole fraction of DMA due to dominating dipole–dipole interactions. At 0.25 *X*_IL_, DMA molecules form continuous domains in all the ILs. The self-aggregation of DMA molecules occurs in TEAH, TPAH, and TBAH ILs. The DMA rich mixtures form the clusters involving DMA molecules. However, the slight distraction of the domain can be observed in TPAH and TBAH mixtures as compared to TEAH. The aggregation of DMA molecules depends on the alkyl chain length of the cation of IL. As we move from lower mole fraction (0.25 *X*_IL_) to higher mole fraction (0.75 *X*_IL_), DMA molecules interact more with IL rather than forming self-aggregated clusters. At lower mole fraction of IL, the breakdown of DMA clusters is difficult due to the lower number of ions. This lower number of ions are unable to form sufficient ion–dipole interactions to destroy the dipole–dipole interactions that are present between DMA molecules. We have observed the effect of alkyl chain length of cation and mole fraction of IL from the visual analysis of simulation boxes. The quantitative assessment of structure will give us more details about the heterogeneity of the mixtures. To address this, we performed a Voronoi based domain analysis.^69^ The domain of liquids consists of building blocks which are subsets. The subsets can be defined in user convenient way, for example, ions of IL or part of cation and part of anion as one subunit. All the atoms present in the system are considered as Voronoi sites. Around each Voronoi site, Voronoi polyhedra are constructed. The borders of polyhedra are defined by using the van der Waals radii of atoms. This approach gives the volumes, surfaces and creates the Voronoi cells for each atom. These atomic Voronoi cells sum up to the cells of each subset. These subsets of the same chemical nature correspond to the same domain if the cells of subsets share a common face. This method allows us to calculate each type of domain present in the liquid (domain count *N*_Dom_). If the *N*_Dom_ is smaller than the total number of subsets present in a system, it represents the aggregation. If the domain count is one, the subsets present in the system are forming a single continuous domain. Voronoi tessellation also allows us to calculate the surface of each subset that is covered by other subsets.

Table 1 depicts the number of domains, volume occupied by each domain, surface covered by each domain and the sphericity (*Q*^Peri^) of the domain. For 0.25 *X*_IL_, the cation subset forms a single continuous domain throughout the system. The formation of continuous domains shows that cations are linked through their alkyl side chains. The anions, on the other hand, show several domains which are almost equal to the number of anions. The hydroxyl anions are very short and are not able to form large aggregates or even small clusters in the ILs. DMA molecules also form a single continuous domain in 0.25 *X*_IL,_ which can be observed from the snapshots in Fig. 8. The number of domains formed by cations at 0.25 *X*_IL_ does not change with the alkyl chain length on the cation. As expected, the domains formed by cations deviate much from spherical nature. The sphericity of cationic domains is in the range of 0.1 to 0.2. Anions facilitate the formation of spherical domains, and DMA molecules show better sphericity than cations. As the cation size increases, they tend to form irregular three-dimensional networks due to the hydrophobic interactions between their alkyl chains. The domains formed by the cations are not spherical when the cation head group is large. At 0.5 *X*_IL_, cations form a continuous single domain, and anions form discrete domains like in 0.25 *X*_IL_. Different behavior can be observed from DMA at 0.5 *X*_IL_. DMA forms 2 to 4 domains in all the three ILs. By increasing the *X*_IL_ from 0.25 to 0.5, the number of domains formed by DMA molecules increases. This indicates the progressive destruction of the network formed by DMA. The domain count in 0.25 *X*_IL_ is 1 for DMA in all the ILs. All the DMA molecules form a continuous network in the 0.25 *X*_IL_ mixtures. The values of *N*_Dom_ at 0.5 *X*_IL_ show utterly different behavior. The increase of *X*_IL_ destroys the network formed by DMA molecules and form more than one domain. An interesting pattern of *N*_Dom_ can be observed at 0.75 *X*_IL_. The domain count of DMA is 11.6 in TEA, which is higher than that of 0.25 and 0.5 *X*_IL_. The DMA clusters formed by dipole–dipole interactions are dissociated into smaller clusters by ions of ILs due to ion–dipole interactions with DMA. Interestingly, the *N*_Dom_ values of DMA increased from 11.6 to 23.0 in TPAH and 37.9 in TBAH at 0.75 *X*_IL_. This increase in *N*_Dom_ is not observed at 0.25 and 0.5 *X*_IL_. This means, after forming sufficient ion–dipole interaction in the liquid, the alkyl chain length plays a role. These results from domain analysis confirm the findings obtained from Fig. 8. Voronoi tessellation also allows us to calculate the information on the neighborhood of each subset and interaction surfaces between the subsets. Table 2 depicts information about the interaction between surfaces. These are the average percentages covered by the different groups of each subset. Cation and anion do not show any particular trend with increasing the alkyl chain length. However, the cation coverage increases with an increase in mole fraction of IL. The anions do not show much coverage around anion as they are apart, and their surfaces do not interact. This finding is consistent with the number of domain counts. Anion coverage around anion does not change with *X*_IL_. Anion coverage around cation decreases with increasing alkyl chain length. Interestingly, the DMA coverage around DMA decreased with increasing alkyl chain length. An increasing microheterogeneity can be seen with alkyl chain length on the cation. For example, 85.25, 66.45, and 54.91 are the percentages of surface coverages by DMA around DMA in TEAH, TPAH, and TBAH, respectively at 0.25 *X*_IL_. This sharp decrease in surface coverage describes that the DMA molecules form larger structural units in ILs with shorter alkyl chain length. This is in good agreement with the conclusions drawn from Fig. 8. This type of behavior can also be observed at 0.5 and 0.75 *X*_IL_. The effect of mole fraction on surface interaction is significant. The cation coverage around cation in TEAH from 0.25 to 0.75 *X*_IL_ increases as expected. Cation coverage around anion is almost constant. Around DMA, cation coverage decreases with the addition of IL. The formation of cation–cation aggregates prevents the DMA interaction with cation. Table S5 and Fig. S9† show the domain analysis based on polar and non-polar parts of the systems. As polar and nonpolar parts form single continuous domains in all the systems, it is difficult to draw any conclusion from these results. The domain count based on individual ions or molecules gives us information about the clustering of ions or molecules. This Voronoi based analysis can be potentially used to draw the quantitative conclusions about the aggregation of particles in a given system.

**Table 1:** Details of domain analysis

Subunit	IL	Domain count (*N*_Dom_)	D-Vol (Å^3^)	D-Surf (Å^2^)	*Q* ^Peri^
**0.25 *X***_**IL**_					
Cation	TEAH	1.024	28 603	13 473	0.20
	TPAH	1.008	41 212	24 687	0.11
	TBAH	1.000	53 529	31 356	0.10
Anion	TEAH	124.641	24.25	46.13	0.82
	TPAH	125.000	24.13	46.00	0.82
	TBAH	125.000	24.20	45.90	0.83
DMA	TEAH	1.600	43 526	7269	0.67
	TPAH	1.011	57 321	21 778	0.19
	TBAH	1.014	56 352	28 957	0.13

**0.5 *X***_**IL**_					
Cation	TEAH	1.000	57 554	24 935	0.16
	TPAH	1.000	81 869	34 639	0.14
	TBAH	1.000	106 197	38 684	0.15
Anion	TEAH	250.000	24.14	45.98	0.82
	TPAH	250.000	24.00	45.44	0.82
	TBAH	250.000	23.86	45.40	0.83
DMA	TEAH	2.480	19 523	8583	0.42
	TPAH	2.250	20 690	14 555	0.36
	TBAH	3.400	14 788	10 375	0.46

**0.75 *X***_**IL**_					
Cation	TEAH	1.000	85 536	29 804	0.18
	TPAH	1.000	122 167	32 510	0.22
	TBAH	1.000	158 912	33 727	0.27
Anion	TEAH	373.500	24.02	45.8	0.82
	TPAH	374.800	23.64	45.3	0.82
	TBAH	375.000	23.70	45.3	0.83
DMA	TEAH	11.600	1680.0	1394.0	0.53
	TPAH	23.000	825.0	780.0	0.55
	TBAH	37.900	495.40	502.8	0.58

**Table 2:** Interaction between surfaces

IL	Cation coverage			Anion coverage			DMA coverage		
	Cation	Anion	DMA	Cation	Anion	DMA	Cation	Anion	DMA
**0.25 *X***_**IL**_									
TEAH	53.894	16.289	29.817	83.796	0.003	16.200	13.339	1.410	85.250
TPAH	40.803	10.312	48.885	75.205	0.000	24.795	31.353	2.181	66.467
TBAH	41.178	7.564	51.258	70.718	0.000	29.282	42.492	2.597	54.911

**0.5 *X***_**IL**_									
TEAH	57.673	16.842	25.486	86.625	0.004	13.370	34.573	3.500	61.900
TPAH	58.330	11.710	29.959	85.698	0.000	14.302	57.968	3.782	38.250
TBAH	63.736	9.016	27.248	68.004	4.070	27.927	87.450	0.000	12.550

**0.75 *X***_**IL**_									
TEAH	66.046	18.000	16.040	91.930	0.005	8.065	66.074	6.474	27.452
TPAH	73.795	12.845	13.360	93.790	0.000	6.210	77.706	4.946	17.347
TBAH	78.870	9.990	11.140	93.820	0.000	6.182	83.537	4.930	11.530

### 3.2 Dynamical properties of the mixtures

**Fig. 9:**
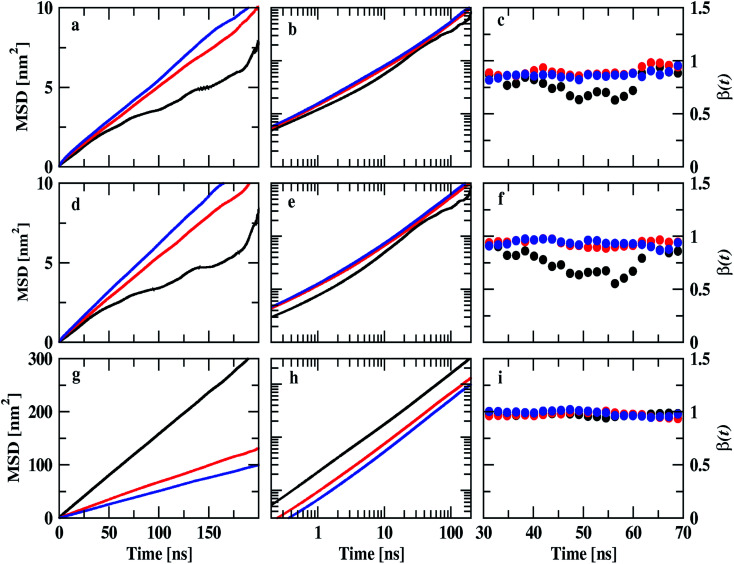
Mean square displacements of ions of IL and DMA molecule at 0.25 mole fraction of IL. Cation, anion, and DMA are shown in the top (a, b and c), middle (d, e, and f) and bottom (g, h and i) rows respectively. Black, red and blue colors represent TEAH, TPAH and TBAH mixtures with DMA. The cations and anions in TEAH show lower diffusion than TPAH and TBAH.

The dynamical behavior of these IL mixtures with DMA was characterized by calculating mean square displacements (MSDs). Diffusion coefficients are calculated from MSD by using the Einstein equation.^71^1
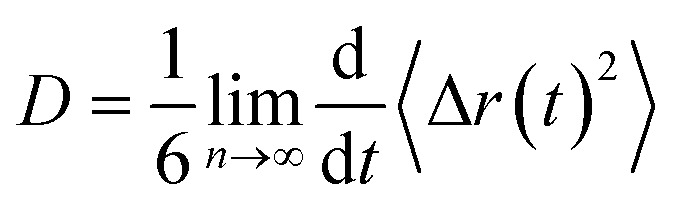
where 〈Δ*r*(*t*)^2^〉 indicates the ensemble-averaged mean square displacements. MSDs were calculated for the center of mass of chosen particles. For ionic liquids, reaching a diffusive regime requires long simulation times. At longer runs, MSDs show linear behavior with time. We performed the simulations for 200 ns for all the trajectories in this study to find the diffusive regions and have converged results. We have also calculated the slope values of the MSD and present in Fig. 9. The slope of the MSD is defined as2
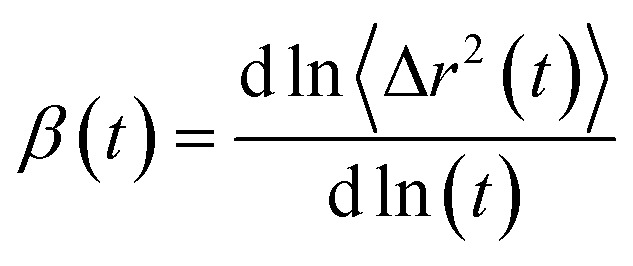


Diffusion coefficients are calculated from the MSDs where *β*(*t*) is near to one and less fluctuating. MSDs corresponding to 0.25 mole fraction of IL are shown in Fig. 9. Other mole fractions of IL are shown in Fig. S10 and S11.† Diffusion coefficients are presented in Table S6.†Fig. 9a shows the MSDs of cations of TEAH, TPAH, and TBAH at 0.25 mole fraction. Fig. 9b and c represent the log–log plot of MSDs and their slope values, respectively. From Fig. 9a, the cation of TEAH has lower diffusion than TPAH and TBAH. The comparison of MSDs of ions of IL with DMA shows that DMA has higher diffusion than both the ions of IL in each mixture. From Fig. 9b and c, the diffusive regime was calculated and found in between 30 to 70 ns time region. At long times the particles of the system showed the Gaussian diffusion which is *β*(*t*) = 1. Table S5† shows the diffusion coefficients of cation, anion, and DMA at three different mole fractions of IL. The self-diffusion coefficient for TEA cation is 0.48 × 10^−7^ cm^2^ s^−1^ for 0.25 mole fraction of IL. The diffusion coefficient decreased almost four times when the concentration of IL is doubled. This nonlinear decrease is due to the slow dynamics of IL + DMA mixture at high concentrations of IL. At higher concentrations of IL, properties of mixtures are close to pure IL. The diffusion coefficient of TEA cation are 0.14 × 10^−7^ and 0.01 × 10^−7^ cm^2^ s^−1^ at 0.5 and 0.75 *X*_IL_, respectively. This is not surprising if we consider the increase in viscosity which is the main factor for the slower dynamics of mixture significantly. Cation and anion exhibit similar diffusion coefficients for all the mixtures studied. A similar diffusion behavior is the result of correlated motion of cations and anions, which is related to ion pair formation. The correlated motion can also be observed at lower mole fractions of IL. Strong electrostatic interactions between counter ions make them stay as an ion pair rather than individual ions. The dissociation of the ion pair is difficult. An interesting pattern can be observed when we consider the effect of alkyl chain length at 0.25 mole fraction of IL. TEA has a diffusion coefficient of 0.48 × 10^−7^ cm^2^ s^−1^ and when we moved to TPA, the diffusion coefficient increases to 0.78 × 10^−7^ cm^2^ s^−1^. This is surprising where the diffusion increases with the molecular mass of cation. The electrostatic interactions which affect correlations between cation and anion are decreased at longer alkyl chain length. This is due to the screening of charge by alkyl chains of cation which hinders the electrostatic attraction between counterions. This can be further observed when the diffusion coefficient increases from TPA to TBA cation. An opposite trend is observed for DMA molecules at 0.25 mole fraction. The diffusion coefficient of DMA in TEAH mixture at 0.25 mole fraction is 26.0 × 10^−7^ cm^2^ s^−1^ and 10.7 × 10^−7^ cm^2^ s^−1^ in TPAH. The diffusion coefficient of DMA decreases more than half when the alkyl chain length is increased on the cation due to the increase in viscosity of mixtures with an increase in the alkyl chain length. A slight decrease in the diffusion coefficient of DMA is observed when cation is changed from TPA to TBA. This observation suggests that at a higher mole fraction of IL, DMA molecules form small aggregates. This feature is already observed from the COM RDFs. At 0.5 mole fraction of IL, cation and anion show similar diffusion coefficients in all three ILs. The values of cation and anion diffusion coefficients are very close. The diffusion coefficient of DMA is much higher than cation and anion. In this case, the diffusion coefficients of cation and anion do not increase with the alkyl chain length. Here, the viscosity factor dominates rather than only ionic interactions. Because of viscous nature at higher *X*_IL_, lower diffusion is observed. At 0.75 mole fraction of IL, the diffusion coefficients are very less and are close. Cation, anion and even DMA also show similar diffusion coefficients at this mole fraction. The high viscosity of IL mixtures causes the slow dynamics at this mole fraction.

Vibrational spectral signature of IL mixtures was probed by calculating the vibrational density of states (VDOs) at low frequencies from velocity–velocity autocorrelation functions. We calculated the vibrational spectra from molecular dynamics simulations by Fourier transform to velocity autocorrelation function (VACFs). The normalized velocity autocorrelation function is defined as^72–75^3
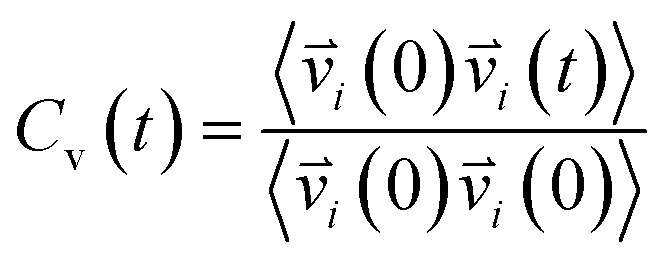

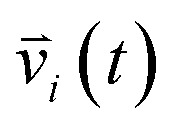
 indicates the velocity of the chosen system at a given time *t*. The angular brackets are for ensemble average to sum over atoms present in the system by considering the different reference times. The Fourier cosine transformation was applied to VACFs to obtain the VDOs.^72–75^4
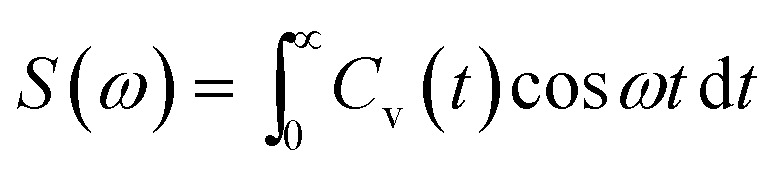


**Fig. 10:**
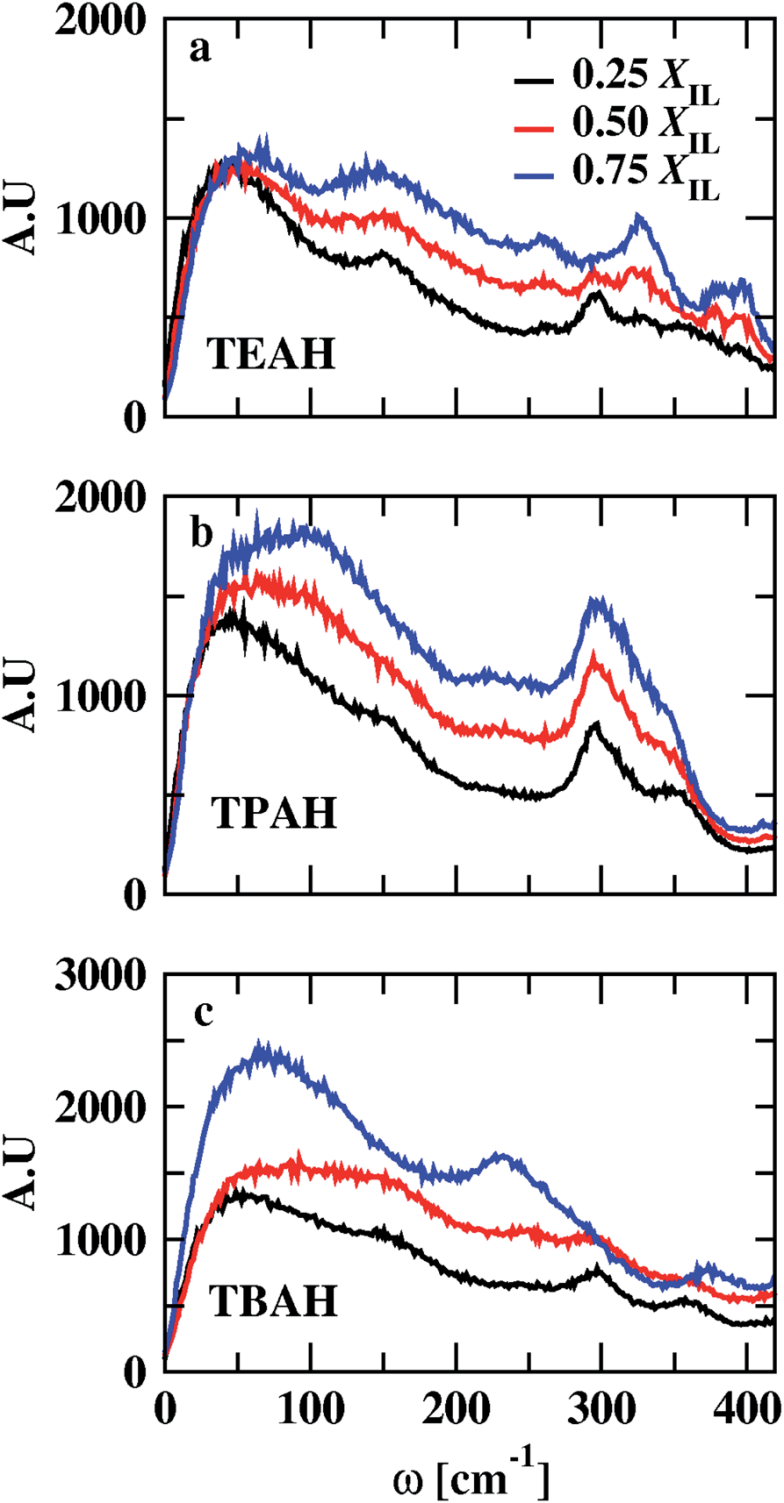
Low-frequency power spectra of the systems at different mole fractions of IL. Black, red, and blue colors represent the 0.25, 0.5 and 0.75 *X*_IL_, respectively. The figures (a–c) indicate the TEAH + DMA, TPAH + DMA and TBAH + DMA mixtures, respectively.

VDOs are shown in Fig. 10. The change in the mole fraction of IL has very less effect on the peak position of VDOs. The low vibrational modes do not change with change in concentration. All the mixtures show peaks around 50 and 300 cm^−1^. Low-frequency bands represent the collisions between chemical entities. Vibrational modes that occur above 180 cm^−1^ correspond to intramolecular vibrational modes of anion.^76–78^ The peak height of VDOs increases with the mole fraction of IL. This is attributed to an increase in collision time between ions and molecules.

### 3.3 Void distribution

**Fig. 11:**
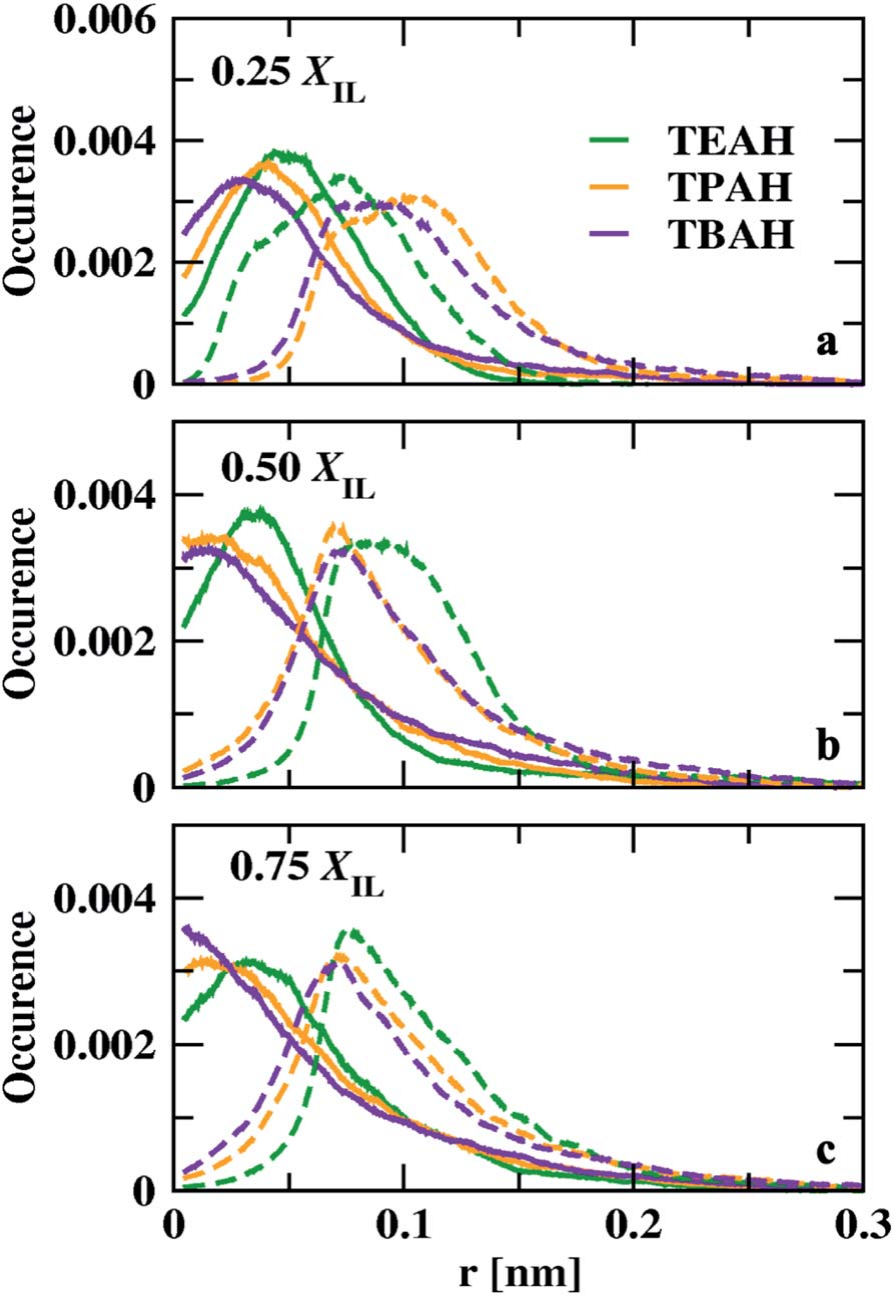
Distribution of interstitial spheres radii for DMA + IL system. Green, orange and, violet colours indicate the TEAH + DMA, TPAH + DMA and, TBAH + DMA, respectively. Solid lines indicate the distribution calculated with van der Waals radius and the dashed lines indicate the half of the van der Waals radius.

We calculated the void distribution in the mixtures following the algorithm and the software developed by Medvedev *et al.*^79^ The interstitial spheres (empty spheres) between four atoms are defined to characterize the voids present in the given system. Voronoi–Delaunay tessellation method was used in this method. This method was used in various fields such as physics, chemistry, and biology.^79–86^ The vertices of the interstitial sphere are present on the four mutually close atoms. This interstitial sphere is connected to Delaunay simplex.^79,87^ The tessellation method used in this version constructed on the surface of the atoms, which can be called weighted^88^ or Voronoi S-tessellation.^79,89^ Unlike the classical approach in which only the centers of atoms^90,91^ were considered, a tangent is drawn on the atomic surface and the distance is measured from the tangent.^92^ The diameters of the atoms are considered from the Lennard-Jones parameters (*σ*) from the molecular dynamics simulations. Recently, Shelepova *et al.*^93^ have compared the void space of an ionic liquid with a neutral mixture of similar-sized molecules. The distribution of voids is shown in Fig. 11. The final trajectories after 200 ns were considered for this analysis. The distribution of voids does not change with time after proper equilibration. Fig. S12† shows void distribution at different time scales. Here, we have calculated the void distribution using two different radii: (1) van der Waals (vdW) radius (2): half of the vdW radius.^94^ When the vdW radius is used, the maximum of the void distribution moved towards the lower distance. All the mole fractions follow a similar order. The size of the voids decreases with the bulkiness of the cation head. The distribution is different in 0.25 mole fraction of IL for half of the vdW radius. 0.50 and 0.75 *X*_IL_s have a similar trend for both vdW and half of the vdW radius. At 0.25 mole fraction, TEAH + DMA show lower void size than the other two mixtures. The lower diffusion of TEAH + DMA mixture at 0.25 mole fraction can be explained from size of the voids. We have calculated the fraction of free volume using the vdW radius for the atoms^95,96^ (Table S7†). As expected the free volume decreased with increasing the alkyl chain length of ILs at all the mole fractions.

## 4. Conclusions

In the present study, we have calculated center of mass (COM) radial distribution functions, combined distribution functions (CDFs), spatial distribution functions (SDFs), domain analysis using Voronoi tessellation method, mean square displacements, diffusion coefficients, and low-frequency spectra to explore the structural and dynamical properties of mixtures of DMA and ILs. We considered DMA molecule in three different ILs TEAH, TPAH, and TBAH at different mole fractions. For each IL, three mole fractions are considered (0.25, 0.5 and 0.75 *X*_IL_). ILs are considered in such a way that the effect of side-chain length on cation can be observed on structural and dynamical properties of mixtures. From the analysis, it was observed that an increasing mole fraction of IL causes the disturbances in the clusters formed by DMA molecules. Due to this, the breakdown of DMA clusters happened at higher mole fractions of IL. The DMA molecules dispersed into the mixture as IL mole fraction increases. Thus, the interactions between DMA molecules and ions of ILs increases with *X*_IL_. Two types of clusters were formed: DMA rich region and IL rich region. The DMA molecules form clusters at DMA rich region. The cations of ILs form a continuous irregular 3D network throughout the box at IL rich region. Hydrophobic interactions between alkyl chains of IL increases with *X*_IL_. This conclusion is consistent with the experimental results. The orientation of cations and anions around DMA molecules change with the addition of alkyl chains on cation, which leads to the microheterogeneity. The dipole–dipole interactions at DMA rich region are enough to form the small clusters. Due to this, DMA molecules can form clusters at DMA rich region. At IL rich region, ion–dipole and ion–ion interactions force DMA molecules to break their self-association. The domain analysis from the Voronoi tessellation method was used to calculate the number of domains formed by the ions and DMA molecules. The alkyl chain length plays a role when it is sufficiently larger. The number of domains formed by the DMA molecules greatly affected by the TBA cation. The surface coverage values reveal that anions do not form any aggregates. The surface of DMA molecules covered by DMA changes with the mole fraction of IL. The rapid decrease in self-diffusion is observed at a higher mole fraction of ILs. At 0.25 mole fraction, even though the molecular weight of TPA is higher than TEA, higher diffusion is found for TPA. The same is observed for TBA at 0.25 mole fraction. This is attributed to a decrease in cation–anion interaction. The decrease in DMA diffusion is attributed to high viscosity and also increase of ion–diploe interactions between ions and DMA molecules. The same is not observed at other mole fractions of ILs due to the highly viscous nature of IL. We have also performed the void distribution analysis to see how the size of the voids changes with the variation on length of the alkyl chain of the cation. The size of the voids decreases with increasing the bulkiness of the cation head. This is one of the reasons to observe the decrease in the diffusion of particles at higher alkyl chain length. The observed results can have a significant impact on the experimental design of IL mixtures for a particular application. The addition or removal of DMA solvent to the IL change the orientation of cation and anion around the DMA molecules. The alkyl chain length of cation also plays a significant role in this regard. Thus, in practice, we can make a mixture of a certain degree of association that serves the desired properties for specific applications. Certain properties can be enhanced or suppressed to the desired level if we know the mechanism of mixing molecular solvents with ILs. With this knowledge, solvents can be prepared in a more particular way for chemical reactions.

## Conflicts of interest

There are no conflicts to declare.

## Supplementary Material

RA-010-C9RA09041J-s001
