# Supplementary material for: Nanostructure domains, voids, and low-frequency spectra in binary mixtures of N,N-dimethylacetamide and ionic liquids with varying cationic size
Source: RSC Adv. 2020 Jan 8;10(3):1811–27. doi: 10.1039/c9ra09041j (PMC9048805; doi:10.1039/c9ra09041j)
Supplement: RA-010-C9RA09041J-s001 [file RA-010-C9RA09041J-s001.pdf]

Electronic Supplementary Material (ESI) for RSC Advances.
This journal is © The Royal Society of Chemistry 2019

Supporting Information

Nanostructure Domains, Voids, and Low-Frequency Spectra in Binary Mixtures of N,N-Dimethylacetamide and Ionic Liquids with Varying Cationic Size

Th. Dhileep N. Reddy and Bhabani S. Mallik*

Department of Chemistry, Indian Institute of Technology Hyderabad, Kandi-502285, Sangareddy, Telangana, India

Table S1. Non-bonding force field parameters for ions and DMA molecule used in this study.

System	Atom	q(e)	σ(Å)	ε(kcal/mol)
TEA	C_1	0.0797	3.475	0.1094
	H_1	0.0715	1.960	0.0157
	N	-0.1884	3.296	0.1500
	C_2	-0.2454	3.475	0.1094
	H_2	0.1066	2.650	0.0157
TPA	C_1	0.0054	3.475	0.1094
	H_1	0.0800	1.960	0.0157
	N	-0.3465	3.296	0.1500
	C_2	0.0644	3.475	0.1094
	H_2	0.0584	1.960	0.0157
	C_3	-0.3395	3.475	0.1094
	H_3	0.1100	2.650	0.0157
TBA	C_1	-0.0454	3.475	0.1094
	H_1	0.0499	1.960	0.0157
	N	0.2226	3.296	0.1500
	C_2	-0.1145	3.475	0.1094
	H_2	0.0735	1.960	0.0157
	C_3	0.0440	3.475	0.1094
	H_3	0.0387	1.960	0.0157
	C_4	-0.2540	3.475	0.1094
	H_4	0.0800	2.650	0.0157
OH	O	-1.2558	3.166	0.1554
	H	0.2558	0.000	0.0000
DMA	N	-0.0005	3.250	0.1700
	O	0.5656	2.960	0.2100

	C1	-0.2294	3.500	0.0660
	C2	0.5345	3.750	0.1050
	C3	-0.3334	3.500	0.0660
	C4	-0.3334	3.500	0.0660
	H1	0.0673	2.500	0.0300
	H2	0.1209	2.500	0.0300

Table S2. Density (g.cm^{-3})of IL + DMA mixtures at different mole fractions**.**

IL	0.25 X_{IL}	0.50 X_{IL}	0.75 X_{IL}
TEAH	0.939	0.959	0.972
TPAH	0.945	0.960	0.966
TBAH	0.947	0.962	0.964

Table S3. First minima and coordination number from Cation-Cation, Anion-Anion, Cation-Anion centre of mass RDFs**.**

IL	X_{IL}	CA		CC		AA		CD		AD		DD	
		FM	CN	FM	CN	FM	CN	FM	CN	FM	CN	FM	CN
TEA	0.25	0.53	2.87	0.94	8.36	0.90	7.74	0.87	5.88	0.60	1.26	0.80	10.91
	0.50	0.53	3.00	0.94	9.15	0.90	8.50	0.87	4.98	0.60	1.04	0.80	7.52
	0.75	0.53	3.14	0.94	10.94	0.90	10.05	0.87	2.88	0.60	0.62	0.80	3.31
TPA	0.25	0.57	2.42	0.93	4.41	0.90	4.24	0.74	4.21	0.61	1.74	0.80	8.83
	0.50	0.57	2.73	0.93	5.98	0.90	5.78	0.74	2.64	0.61	1.03	0.80	5.04
	0.75	0.57	2.90	0.93	7.61	0.90	7.21	0.74	1.21	0.61	0.48	0.80	2.16
TBA	0.25	0.61	2.14	0.90	2.89	0.90	2.94	0.76	4.76	0.62	2.07	0.80	7.35
	0.50	0.61	2.52	0.90	4.21	0.90	4.43	0.76	2.51	0.62	1.07	0.80	3.87
	0.75	0.61	2.74	0.90	5.09	0.90	5.43	0.76	1.05	0.62	0.43	0.80	1.61

CA=cation-anion, CC=cation-cation, AA=anion-anion, CD=cation-DMA, AD=anion-DMA, DD=DMA-DMA, FM=first minimum, CN=coordination number

Table S4. Ion pair lifetimes (ns) of IL + DMA mixtures at different mole fractions.

IL	0.25 X_{IL}	0.50 X_{IL}	0.75 X_{IL}
TEAH	7.31	8.56	10.65
TPAH	8.78	12.92	14.80
TBAH	10.65	23.50	28.17

Table S5. Domain count according to polar and non polar definitions.

Subunit	IL	Domain Count	D-Vol(Å^3)	D-Surf(Å^2)	Q^{peri}
0.25 X_{IL}					
Nonpolar					
	TEAH	1.290	15537	17974	0.14
	TPAH	2.414	16345	16881	0.36
	TBAH	1.290	37032	33035	0.15
Polar					
	TEAH	1.002	72803	20632	0.26
	TPAH	1.000	71725	31316	0.14
	TBAH	1.000	70601	38111	0.10
0.5 X_{IL}					
Nonpolar					
	TEAH	1.117	33429	37588	0.09
	TPAH	1.110	56686	48839	0.09
	TBAH	1.001	84655	56136	0.07
polar					
	TEAH	1.002	66432	39773	0.09
	TPAH	1.000	65641	51574	0.06
	TBAH	1.220	59237	52071	0.11
0.75 X_{IL}					
Nonpolar					
	TEAH	1.014	52094	54379	0.05
	TPAH	1.002	89087	62974	0.06
	TBAH	1.000	126781	64353	0.08
polar					
	TEAH	1.003	60598	54663	0.05
	TPAH	1.000	60449	62994	0.04
	TBAH	1.002	59076	63803	0.05

Table S6. Diffusion coefficients of ions and DMA molecules at different mole fractions in three ILs. ($\times 10^{-7}$ cm^2s^{-1})

XIL	Cation	Anion	DMA
TEAH			
0.25	0.48(±0.005)	0.44(±0.007)	25.97(±0.006)
0.50	0.14(±0.006)	0.17(±0.001)	2.69(±0.004)
0.75	0.01(±0.001)	0.02(±0.001)	0.03(±0.001)
TPAH			
0.25	0.78(±0.006)	0.85(±0.004)	10.73(±0.050)
0.50	0.06(±0.001)	0.06(±0.001)	0.89(±0.080)
0.75	0.01(±0.001)	0.01(±0.001)	0.08(±0.001)
TBAH			
0.25	0.88(±0.003)	0.99(±0.006)	8.32(±0.038)
0.50	0.09(±0.0001)	0.10(±0.001)	1.23(±0.009)
0.75	0.02(±0.001)	0.02(±0.001)	0.22(±0.001)

Table S7. Fraction of free volume in IL + DMA mixtures at different mole fractions**.**

IL	0.25 X_{IL}	0.50 X_{IL}	0.75 X_{IL}
TEAH	0.847	0.840	0.833
TPAH	0.841	0.832	0.827
TBAH	0.836	0.827	0.823

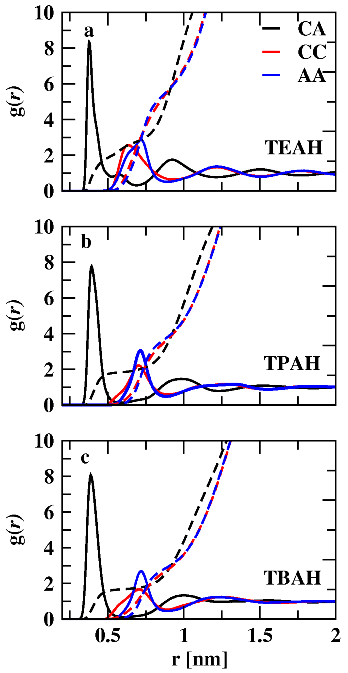

Figure S1. COM radial distribution functions for 0.5 mole fraction between cation-anion (CA), cation-cation (CC) and anion-anion (AA).

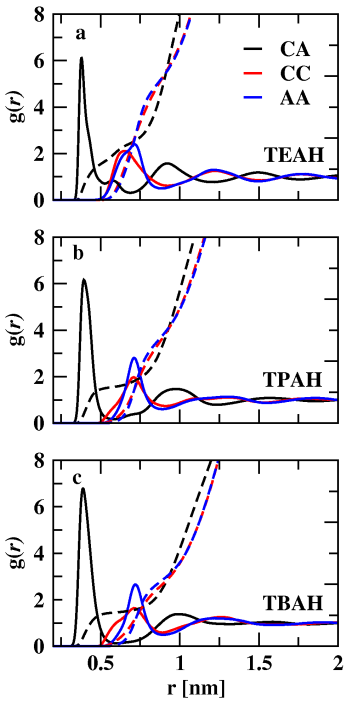

Figure S2. COM radial distribution functions for 0.75 mole fraction between cation-anion (CA), cation-cation (CC) and anion-anion (AA).

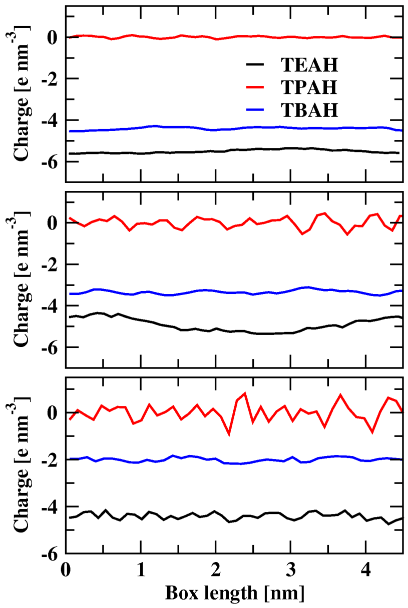

Figure S3. Charge density distributuion along the z-axis in IL + DMA mixtures.

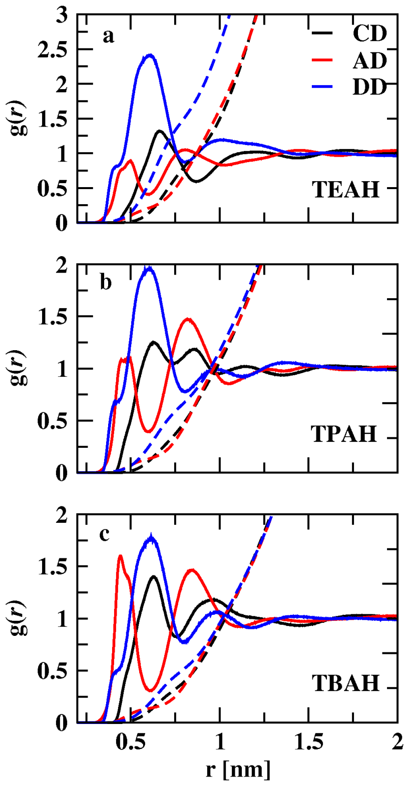

Figure S4. COM radial distribution functions for 0.5 mole fraction between cation-DMA (CD), anion-DMA (AD) and DMA-DMA (AA).

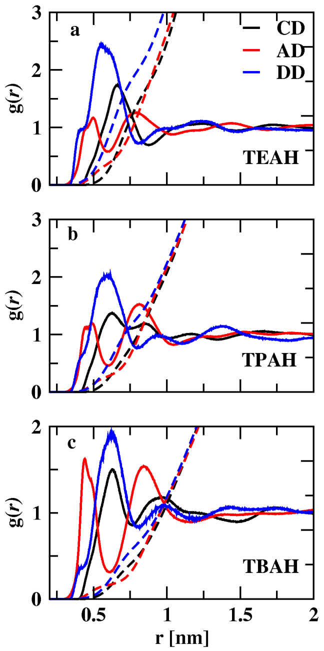

Figure S5. COM radial distribution functions for 0.75 mole fraction between cation-DMA (CD), anion-DMA (AD) and DMA-DMA (AA).

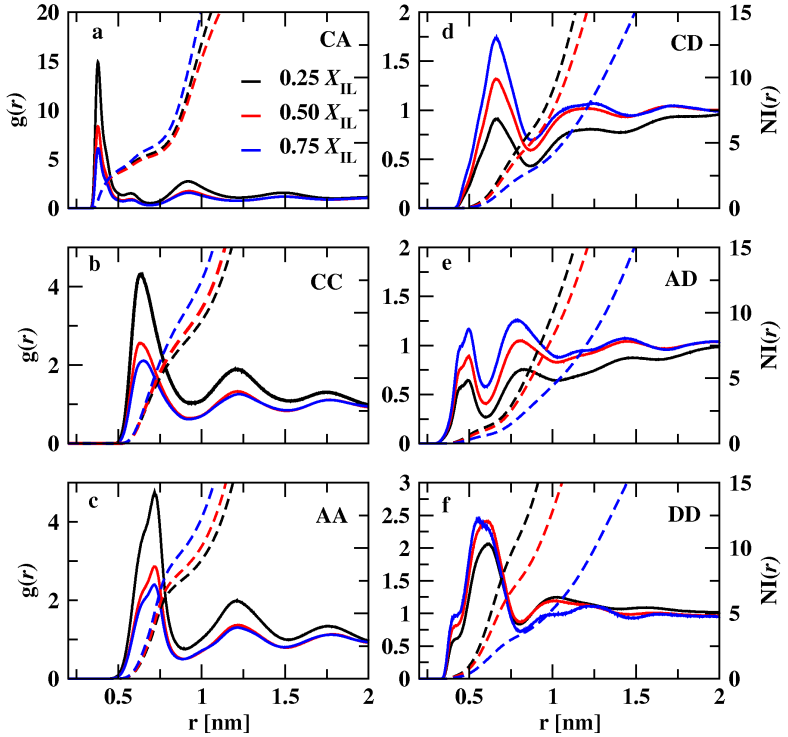

Figure S6. Comparison of COM radial distribution functions between possible pairs with changing mole fraction of TEAH in TEAH+DMA mixture.

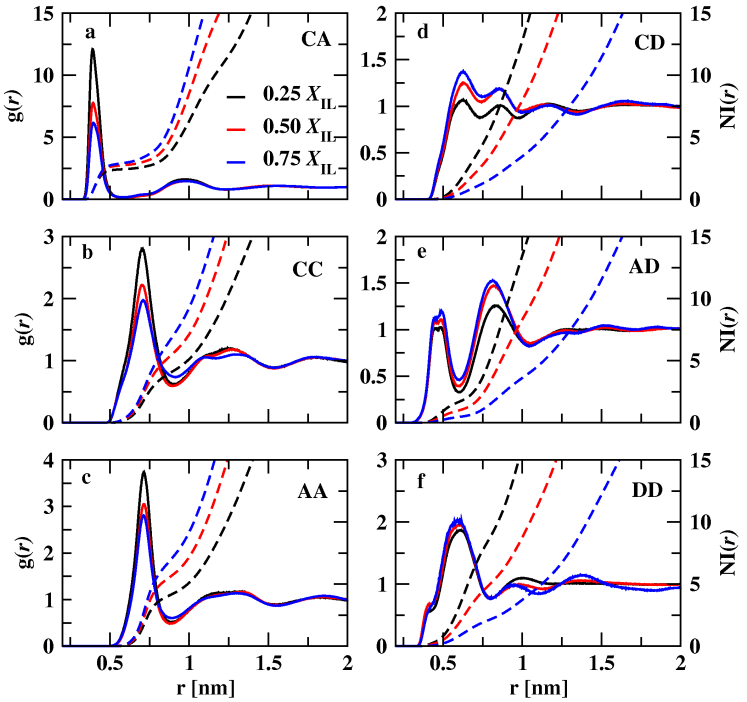

Figure S7. COM radial distribution functions between all the possible pairs to see the effect of mole fraction of TPAH in mixture.

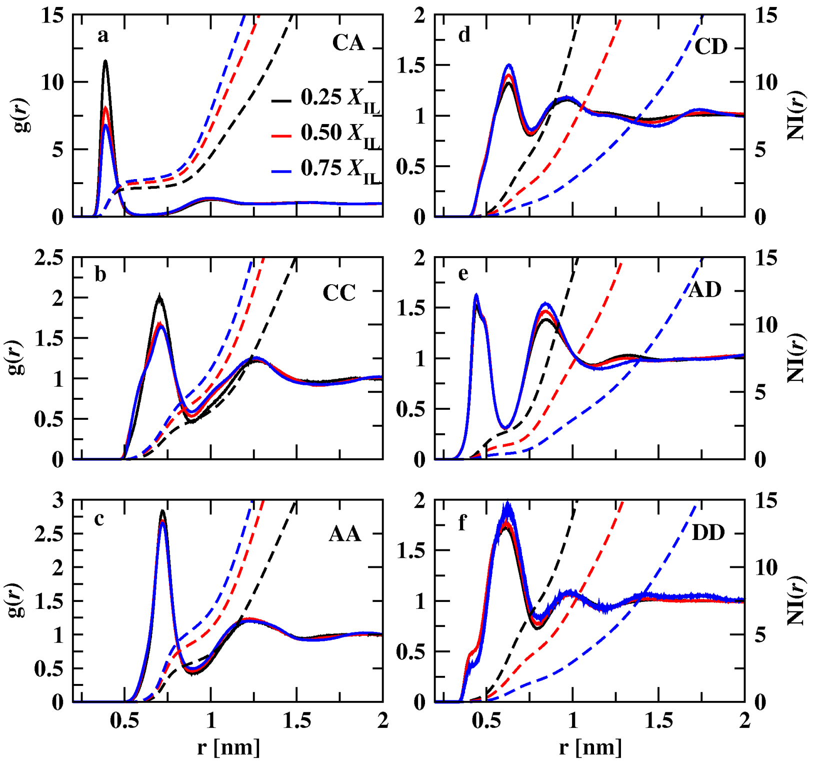

Figure S8. COM radial distribution functions between different pairs that are possible in TBAH + DMA mixture with changing mole fraction of IL.

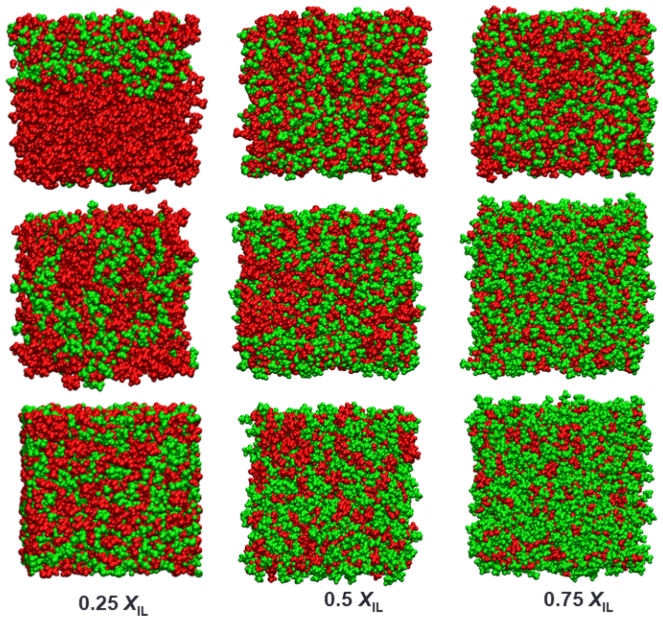

Figure S9. Polar and nonpolar network formation of all the systems studied in this systems. Top, middle and bottom rows show the mixtures of DMA with TEAH, TPAH, and TBAH, respectively. Red color indicates the polar network and green color indicates nonpolar network. DMA, anions, nitrogen atoms of cation and methylene groups that are attached to nitrogen atoms of cation are considered as polar part and methyl and methylene groups that are not attached to nitrogen atom of cation are considered as nonpolar part.

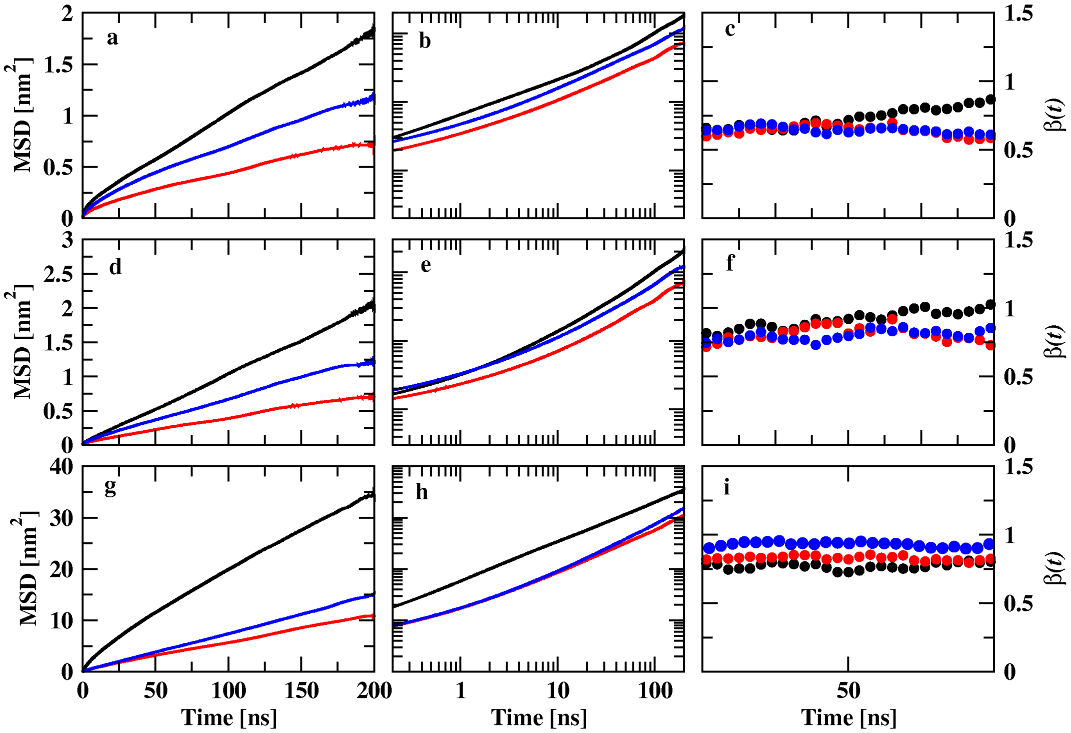

Figure S10. Mean square displacements corresponding to 0.5 mole fraction of IL. Colors are according to Figure 8.

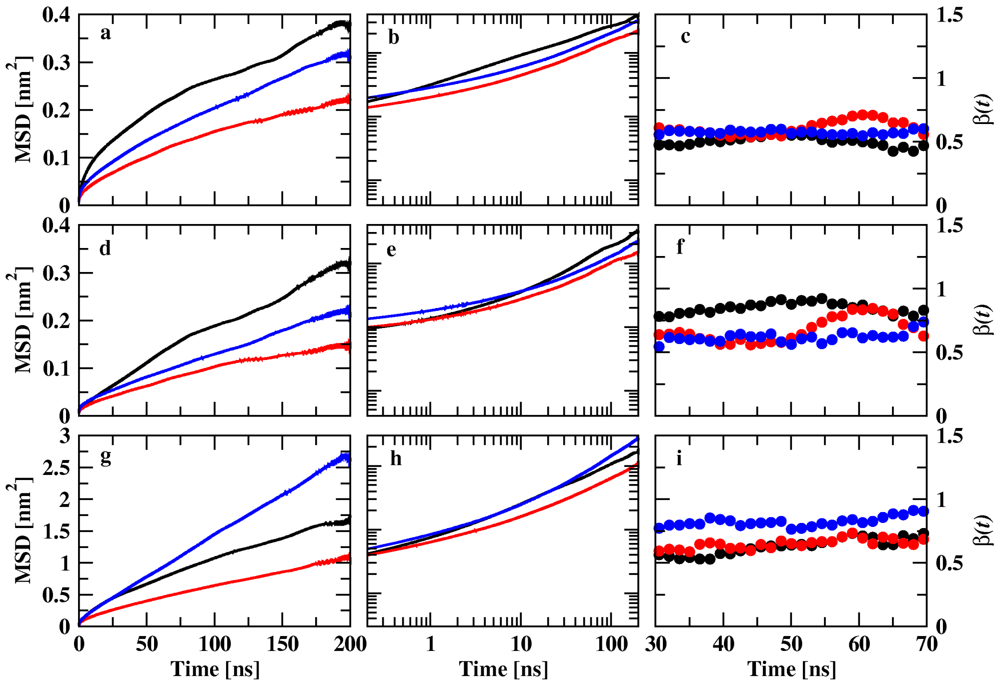

Figure S11. Mean square displacements corresponding to 0.75 mole fraction of IL. Colors are according to Figure 8.

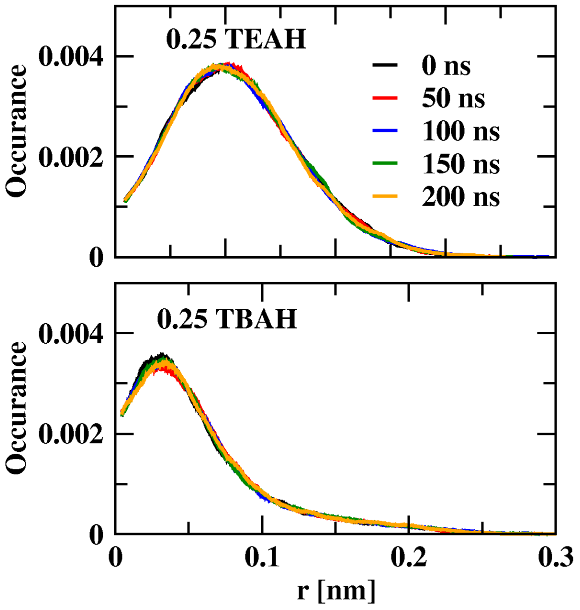

Figure S12. Distribution of interstitial spheres at different times in DMA + IL systems.

Optimized structures of ions in XYZ format

TEA

```
N      0.000   0.000   0.000
C      0.000   1.225   0.915
H     -0.875   1.118   1.553
H      0.875   1.118   1.553
C      0.000   2.579   0.226
H     -0.887   2.748  -0.384
H      0.000   3.341   1.008
H      0.887   2.748  -0.384
C     -0.000  -1.225   0.915
H     -0.875  -1.118   1.553
H      0.875  -1.118   1.553
C     -0.000  -2.579   0.226
H      0.887  -2.748  -0.384
H     -0.000  -3.341   1.008
H     -0.887  -2.748  -0.384
C     -1.225   0.000  -0.915
H     -1.118  -0.875  -1.553
H     -1.118   0.875  -1.553
C     -2.579   0.000  -0.226
H     -3.341   0.000  -1.008
H     -2.748  -0.887   0.384
H     -2.748   0.887   0.384
C      1.225  -0.000  -0.915
H      1.118  -0.875  -1.553
C      2.579  -0.000  -0.226
H      2.748  -0.887   0.384
H      3.341  -0.000  -1.008
H      2.748   0.887   0.384
H      1.118   0.875  -1.553
```

TPA

```
N       0.000   0.000   0.000
C       0.000   1.263   0.864
H       0.977   1.299   1.345
H      -0.055   2.097   0.168
C      -1.106   1.387   1.907
```

H	-1.072	0.554	2.613
H	-2.091	1.370	1.436
C	-0.945	2.704	2.677
H	-1.014	3.567	2.012
H	-1.729	2.801	3.428
H	0.017	2.751	3.194
C	1.263	-0.000	-0.864
H	2.097	0.055	-0.168
H	1.299	-0.977	-1.345
C	1.387	1.106	-1.907
H	1.370	2.091	-1.436
H	0.554	1.072	-2.613
C	2.704	0.945	-2.677
H	2.801	1.729	-3.428
H	3.567	1.014	-2.012
H	2.751	-0.017	-3.194
C	-1.263	0.000	-0.864
H	-2.097	-0.055	-0.168
C	-0.000	-1.263	0.864
H	0.055	-2.097	0.168
H	-0.977	-1.299	1.345
C	1.106	-1.387	1.907
H	1.072	-0.554	2.613
H	2.091	-1.370	1.436
C	0.945	-2.704	2.677
H	1.729	-2.801	3.428
H	-0.017	-2.751	3.194
H	1.014	-3.567	2.012
C	-1.387	-1.106	-1.907
H	-1.370	-2.091	-1.436
H	-0.554	-1.072	-2.613
C	-2.704	-0.945	-2.677
H	-2.751	0.017	-3.194
H	-2.801	-1.729	-3.428
H	-3.567	-1.014	-2.012
H	-1.299	0.977	-1.345

TBA

N	-0.111	-0.064	-0.055
C	-0.854	1.242	-0.341
H	-1.448	1.449	0.549
H	-0.084	2.006	-0.410
C	-1.742	1.271	-1.582
H	-2.502	0.490	-1.525
H	-1.155	1.087	-2.486
C	-2.449	2.633	-1.726
H	-3.185	2.533	-2.527
H	-3.023	2.842	-0.817
C	0.595	0.060	1.296
H	-0.190	0.281	2.018

H	0.983	-0.931	1.525
C	1.705	1.100	1.406
H	1.323	2.104	1.202
H	2.499	0.905	0.681
C	2.309	1.080	2.819
H	1.520	1.271	3.554
H	2.694	0.078	3.035
C	0.894	-0.313	-1.182
H	0.299	-0.440	-2.084
C	-1.077	-1.250	0.008
H	-0.469	-2.110	0.278
H	-1.430	-1.404	-1.011
C	-2.261	-1.134	0.963
H	-2.889	-0.278	0.705
H	-1.924	-0.987	1.991
C	-3.114	-2.411	0.900
H	-3.448	-2.574	-0.130
H	-2.493	-3.275	1.163
C	1.839	-1.499	-1.016
H	1.281	-2.434	-0.924
H	2.443	-1.394	-0.112
C	2.777	-1.602	-2.229
H	3.344	-0.671	-2.330
H	2.180	-1.699	-3.142
H	1.460	0.611	-1.288
C	-1.523	3.809	-2.042
H	-2.104	4.717	-2.209
H	-0.824	4.026	-1.230
H	-0.940	3.621	-2.947
C	-4.326	-2.349	1.829
H	-4.912	-3.267	1.764
H	-4.023	-2.223	2.871
H	-4.984	-1.517	1.568
C	3.742	-2.782	-2.121
H	3.204	-3.731	-2.058
H	4.395	-2.830	-2.994
H	4.378	-2.698	-1.236
C	3.428	2.107	2.990
H	3.065	3.123	2.818
H	3.838	2.068	4.000
H	4.249	1.921	2.292

OH

O	0.000	0.000	0.107
H	0.000	0.000	-0.859

DMA

C	1.778	0.803	-0.000
H	1.702	1.441	-0.884
H	2.749	0.316	0.000
H	1.702	1.441	0.884

C	0.725	-0.294	-0.000
N	-0.593	0.083	-0.000
O	1.060	-1.471	0.000
C	-1.628	-0.939	0.000
H	-2.261	-0.841	0.888
H	-2.262	-0.840	-0.887
H	-1.155	-1.916	-0.001
C	-1.072	1.452	0.000
H	-1.688	1.644	0.886
H	-0.249	2.160	-0.001
H	-1.689	1.643	-0.884
